# Flexible On-the-Fly Active Learning for Molecular Infrared Spectra

**DOI:** 10.1021/acs.jctc.6c00756

**Published:** 2026-09-28

**Authors:** Giulio Benedini, Matti Hellström, Lucas Visscher

**Affiliations:** † 64333 Software for Chemistry & Materials BV, De Boelelaan 1109, Amsterdam 1081 HV, The Netherlands; ‡ 1190 Vrije Universiteit Amsterdam, De Boelelaan 1108, Amsterdam 1081 HZ, The Netherlands

## Abstract

Infrared spectroscopy serves as an indispensable analytical tool across materials science, pharmaceutical development, and the life sciences. Computational insights derived from first-principles methods enhance the interpretation of vibrational spectra, yet their application remains hindered by high computational cost. Machine-learned interatomic potentials offer a route to accelerate these predictions by multiple orders of magnitude without sacrificing ab initio accuracy. Here, we present an on-the-fly active learning framework for the automated training of such potentials specifically tailored to infrared spectral prediction. Our approach employs a multitask acquisition strategy, in which model performance is evaluated iteratively on designated objectives, avoiding the dependence on fixed training sets. We demonstrate that this multitask scheme creates interatomic potentials that exhibit robustness and fidelity in complex workflows for calculating infrared spectra.

## Graphical abstract

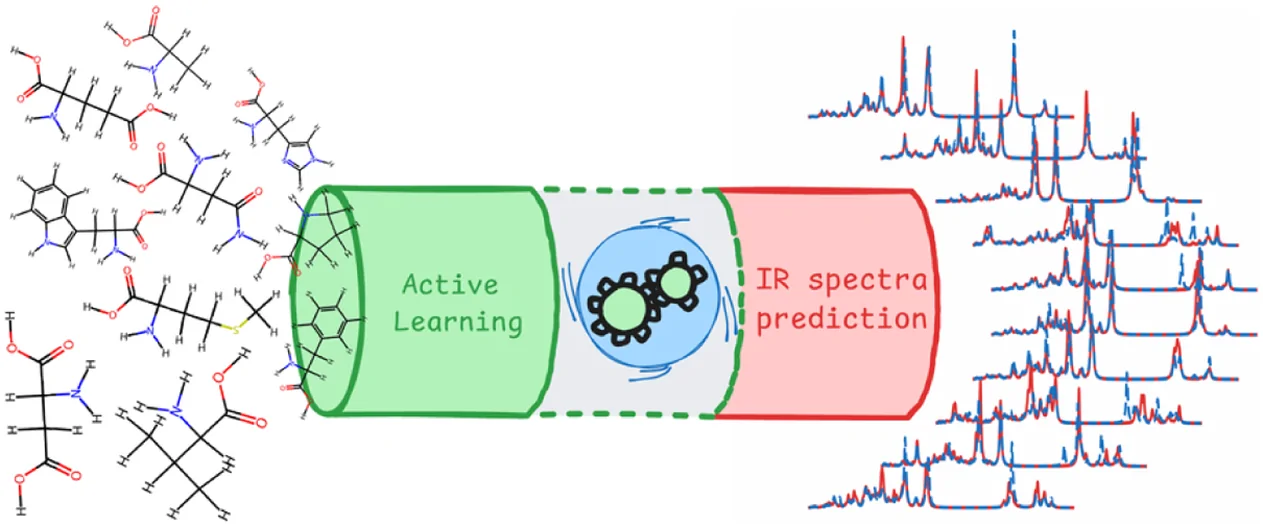

## 1 Introduction

Spectroscopic analysis in the mid-infrared (IR) region has been extensively employed across diverse domains, including industrial process monitoring^1,2^ materials science,^3^ and medical diagnostics.^4–8^ Its capacity to furnish precise quantitative information regarding chemical composition, structural parameters, and molecular properties of both individual molecules and bulk materials underpins its widespread utility.^9^ As a result, there is a strong scientific and commercial motivation to create accurate models for predicting vibrational spectra in the mid-IR range.^10^ Such simulations not only facilitate the interpretation of experimental spectra but also enable the rational design of novel molecular and material systems with tailored infrared characteristics.

From a computational standpoint, density functional theory (DFT) has emerged as the method of choice for predicting vibrational properties and IR spectra, offering a favorable balance between accuracy and cost.^11^ Nevertheless, DFT remains prohibitively expensive for realistically sized systems and for the long simulations required to capture anharmonic and dynamical effects.^12,13^ Lower-cost quantum-chemical methods, including semiempirical approaches such as density-functional tight binding, provide an important alternative route to faster IR workflows.^14,15^ Their advantage is the immediate reduction in the cost per electronic-structure calculation, but this comes at the expense of accuracy. To mitigate this loss, machine-learning interatomic potentials (MLIPs) have shown remarkable success in reproducing potential-energy surfaces at near first-principles accuracy while dramatically reducing computational cost.^16,17^

Spectroscopic applications, however, impose additional requirements on model outputs. Beyond energies and forces (i.e., gradients), one must also predict observables that determine IR intensities, most notably the electric dipole moment. Several MLIPs that jointly predict energies, forces, and dipoles (hereafter MLIP-EFD) are now available, including FieldSchNet,^18^ MACE-EFD,^19^ and AIMNet2.^20^ These models provide all necessary quantities for IR simulations, in contrast to the “two-model” approach (MLIP-EF+D) in which one model predicts energies/forces and a second predicts dipoles.^21–24^

Training MLIP-EFD models presents its own set of challenges. Large, general-purpose data sets^25–27^ promise broad chemical, conformational, and configurational coverage, motivating the development of *universal* MLIPs (uMLIPs).^20,23,28^ Such efforts need to be constantly kept up to date with rapid changes in architectures and the steady stream of improved data sets, which render earlier uMLIPs less effective. Conversely, training system-specific models requires assembling diverse and representative data and computing expensive DFT (or higher level) reference labels. Active learning (AL)^29,30^ mitigates these costs by iteratively identifying and labeling the most informative configurations, invoking reference calculations only when needed. Ultimately, these two approaches can converge with the usage of the uMLIPs as starting point for the AL fine-tuning step.^31^

The purpose of this work is to investigate on-the-fly^32^ active learning strategies for training machine-learned interatomic potentials (MLIPs) capable of predicting infrared spectra with high fidelity. On-the-fly active learning offers a particularly appealing paradigm because it dynamically selects the most informative configurations for reference quantum-mechanical calculations during the simulation itself, thus reducing redundant data generation and improving model generalization. The objective is to assess how such adaptive sampling schemes can accelerate the development of MLIPs that combine the accuracy of first-principles methods with the computational efficiency required for large-scale infrared simulations. The complete procedure described in this paper is depicted in Figure 1.

**Figure 1:**
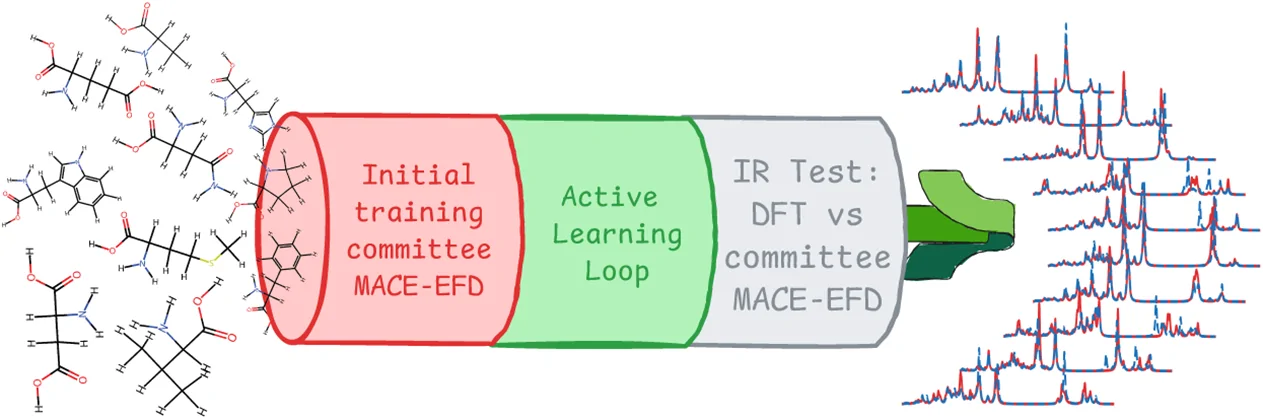
Complete procedure followed in this work.

In this work, we investigate this question for a chemically diverse set of biomolecular building blocks comprising the 20 proteinogenic amino acids and 10 representative dipeptides. These systems provide a challenging test set because of their conformational flexibility and rich vibrational structure. All MLIP models presented in this study are based on the MACE architecture,^33^ a message-passing equivariant MLIP framework that has demonstrated high accuracy for molecular systems.^34^

The implementation section of this paper is organized as follows. In Subsection [Sec sec2.1] we describe the implementation of the AL workflow, in Subsection [Sec sec2.2] the associated settings, while Subsection [Sec sec2.3] provides details on specific components tailored to IR spectra. In Subsection [Sec sec2.4] the chosen MLIP architecture is described and in Subsection [Sec sec2.5] the reference level of theory. Subsection [Sec sec2.6] introduces the test sets employed to assess the performance of the AL workflow, while Subsection [Sec sec2.7] presents the methodologies adopted for this assessment. In the Results section, we address the following question: can one construct an AL workflow for IR spectra that is both robust and data efficient? We first inspect the robustness requirement in Subsection [Sec sec3.1]. By robustness, we mean achieving an accurate MLIP-EFD over extensive regions of the potential energy surface (PES). Then, in Subsection [Sec sec3.2] we discuss the accuracy of the resulting models in the context of spectral interpretation. Finally, we consider the data efficiency requirement in Subsection [Sec sec3.3] in which different AL workflows, all possible with our implementation, are compared.

## 2 Implementation Details

### 2.1 Active Learning Workflow

Most of the work on AL for IR has been applied to MLIP-EF+D models.^21,35,36^ Our active learning workflow follows a similar paradigm: a committee of MLIP models (cMLIP) performs a task (e.g., molecular dynamics); selected structures are evaluated with DFT-level reference calculations; and the cMLIP is retrained until the uncertainty estimate converges (in Figure S1). Our implementation adopts a pool-based, multitask active learning approach with a committee of MLIP-EFD models (cMLIP-EFD), and it includes validity and accuracy criteria. The workflow is shown in Figure 2 and extends the simple active learning available in the Amsterdam Modeling Suite (AMS).^37^ We define for each molecule, inside a set of *active systems*, a *molecule journey* that consists of *tasks*, *validity checks*, *frame getters*, *accuracy checks*, *pre/postfilters*, and *cMLIP training*. The AL loop proceeds as follows:

**Figure 2:**
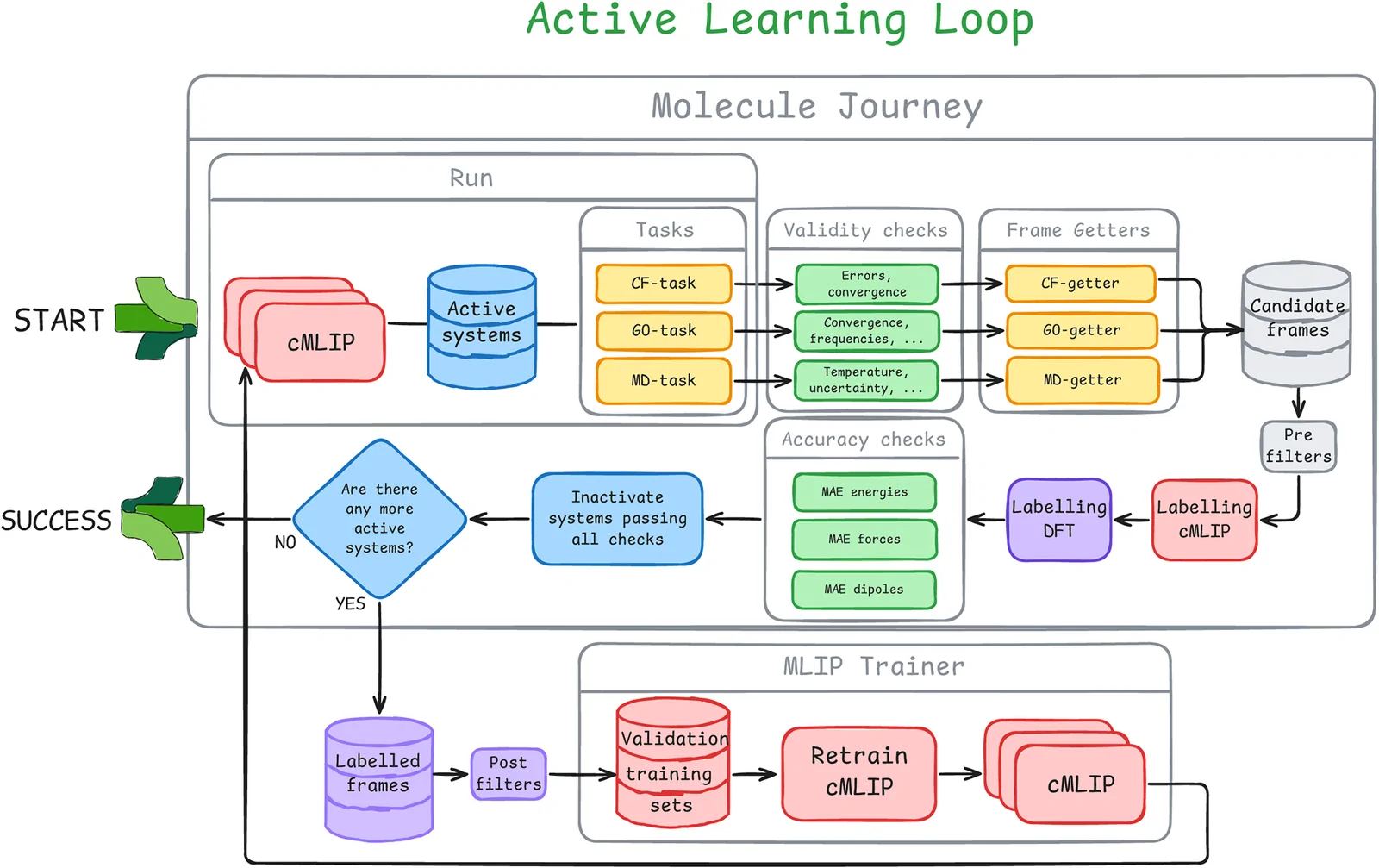
Scheme of the active learning workflow. In red, the cMLIP-related components (cMLIP = committee of machine learning interatomic potentials). In blue, the active systems and the related decision-making step to inactivate systems passing all checks, as well to end the active learning workflow. In yellow, the tasks and frame getters, which are crucial components for checking the reliability of cMLIP and for selecting frames for retraining. In green, the validity and accuracy checks used in the decision-making. In gray, selected frames and prefilters. Finally, depicted in violet are labeling at DFT level, labeled frames, and postfilters.

#### 2.1.1 Initialization

To set up the AL loop, we first add molecules, or more generally atomistic systems, to the *active systems* set. This is the set of systems on which we evaluated the cMLIP-EFD.

#### 2.1.2 Tasks and Frame Getters

In the AL loop, for each *active system,* we run a configurable set of tasks with the cMLIP-EFD. We subsequently run the *frame getter* (orange in Figure 2) to obtain *candidate frames* of these active systems that can be used to assess and retrain the cMLIP-EFD. In this paper, we implemented the following *tasks* and *frame getters* relevant for IR spectra calculations:

- Geometry optimization (GO-*task*) was used to check whether the cMLIP-EFD can find local minima of the PES and predict harmonic IR spectra. Specific to this task is the GO-*getter*, which collects from optimization trajectory frames and performs normal-mode sampling^38^ on modes computed with the current cMLIP-EFD model.
- Molecular dynamics (MD-*task*) to assess the model’s ability to explore higher-energy regions of the PES and assess the anharmonicity in the vicinity of minima. The corresponding MD-*getter* selects frames from the MD trajectory informed by the model uncertainty.
- Conformer search (CF-*task*) with the corresponding CF-*getter* generates and samples multiple low-energy PES minima.

#### 2.1.3 Filters and Frame Labeling

To the obtained set of *candidate frames,* we apply *prefilters* to remove unnecessary or undesirable frames. These filters only consider the geometries of the selected frames. By default, we use a filter to remove structures in which any pair of atoms is separated by less than 0.7 Å. After this filtering, the remaining frames are queued for labeling at the DFT level of theory. As the cMLIP-EFD is not yet run for all frames (for instance, the ones generated by the normal mode sampling), we also label the frames with the cMLIP-EFD for later application of *accuracy checks*.

#### 2.1.4 Validity and Accuracy Checks

With all labels for the new frames in place, we combine *validity checks* and *accuracy checks* to decide which molecules in the *active systems* should be retained for the next series of tasks or can be flagged as inactive. The *validity checks* component ensures simulation stability and steers the decisions made by the *frame getters*; this includes common-sense checks (e.g., detecting molecular explosions, implosions, or sudden energy jumps in an MD trajectory) as well as uncertainty-based measures (e.g., the standard deviation of predicted properties across the cMLIP). *Accuracy checks*, in contrast, evaluate how well the cMLIP reproduces the properties of newly sampled structures. Combining the two kinds of checks yields stricter and more reliable convergence conditions for each molecule of interest.

#### 2.1.5 Filters and cMLIP Training

If the cMLIP is not converged for all molecules, the cMLIP is retrained on the expanded *validation and training sets* with the newly labeled data. The data splitting is performed by grouping by origin, as determined by the frame getter, and 10% of each group is allocated to the validation set, thereby preserving the representativeness of the validation subset as more data are added. Before this step, *postfilters* can be applied based on both geometry and labels. In the current work, we only used one *postfilter*, which excludes frames with a maximum force greater than 10 eV/Å.

#### 2.1.6 AL Loop Convergence

When no *active systems* remain, the AL loop is terminated.

This extended workflow formalizes common active learning strategies while retaining flexibility and modularity. Looking beyond the current application to simulate IR spectra, it allows for easy extension with new tasks while maintaining an overall software architecture.

### 2.2 Task and Frame Getter Settings

The tasks during the AL loop were performed using AMS^37^ and its interface to Python-based engines such as the current cMLIP-EFD.

The GO-*task* consists of a geometry optimization followed by harmonic frequency calculations with the cMLIP-EFD. We employed a Quasi-Newton optimizer^39–41^ with convergence criteria set to the AMS ″Good″ default (energy changes smaller than 0.027 meV, a maximum gradient of 0.27 meV/Å, and a maximum displacement of 10^–3^ Å). At the obtained local minima, if the geometry optimization converged, modes and frequencies of vibrations are obtained by diagonalization of the mass-weighted force constant matrix (**W**):

1 $$W_{ij}=M^{-\frac{1}{2}}\frac{\partial^{2}E}{\partial R_{i}\partial R_{j}}M^{-\frac{1}{2}}$$

with *M* being the diagonal matrix of the atomic masses, *R* the atomic positions, and *E* the molecular energy. The calculation and diagonalization of **W** are performed for each of the three MLIP-EFD committee members separately. In addition, the average spectrum is computed by diagonalizing also the averaged Hessian matrix. In the numerical calculation of the Hessian, displacements of 0.005 bohr are used, while the IR absorption coefficients **A** are evaluated for each normal frequency *ν̃* in the double-harmonic approximation:^42,43^

2 $$A_{\mathrm{\nu ̃}}=\frac{1}{4\pi \epsilon_{0}}\frac{N_{A}\pi }{3c^{2}}\overset{\left(x,y,z\right)}{\underset{\alpha }{\sum }}{\left(\sum_{j}^{3N_{at}}\frac{\partial \mu_{\alpha }}{\partial R_{j}}\frac{\partial R_{j}}{\partial Q_{j,\mathrm{\nu ̃}}^{\left(m\right)}}\right)}^{2}$$

with *N*_*A*_ being Avogadro’s constant, c the speed of light, **μ** the molecular electric dipole moment, *j* indexing Cartesian nuclear displacements, and **Q** ^(*m*)^ providing the transformation to the mass-weighted (*m*) normal coordinates.

After the GO-*task*, the GO-*getter* protocol is employed. It consists of collecting up to two frames from the geometry-optimized trajectory (the last and the middle one). In addition, if the geometry optimization converged, we generated an extra five samples with normal-mode sampling^38^ at 300 K on modes computed with the current cMLIP-EFD model.

The MD-*task* consists of a constant particle number, volume, and temperature (NVT) molecular dynamics simulation at 300 K for 5 ps, with a Nosé–Hoover thermostat,^44,45^ a damping coefficient of 200 fs, and a time step of 0.5 fs. We compute early stop conditions (called exit conditions in AMS) for this simulation based on a maximum uncertainty in the gradients of 800 meV/Å, and an uncertainty in the energy of 27 meV/atom. The uncertainty is defined as the standard deviation of the three committee members’ predictions. We added an additional early stop condition when any two atoms approach each other more closely than 0.7 Å. These correspond to the default criteria of the simple active learning module of AMS.^37^ When any of the early stop conditions is met, the simulation is preemptively stopped. Occurrences of such events and the conditions that led to the early stop are collected for investigation by the *validity checks* later in the AL loop.

After MD-*task*, the MD-*getter* protocol is employed. It consists of two consecutive selection criteria to the frames produced by the MD trajectory: selection of the top 25% of frames by uncertainty in energy, and then farthest-point sampling based on the MD timestamp collecting up to five frames. If there is an early stop of the MD that results in less than five frames, then only one frame will be collected. This procedure retains the most uncertain and least-correlated frames.

For the CF-*task*, we use the AMS^37^ implementation for conformer generation. Five candidate conformers are generated with RDKit,^46^ the geometries are optimized using only the cMLIP-EFD, and then duplicates are filtered out. Note that this number of conformers is not sufficient for an exhaustive search, but it is enough for creating additional diversity in the frame generation.

Following CF-*task*, the CF-*getter* protocol was applied. If the initial geometry optimization performed during the CF-*task* fails to converge or if the CF-*task* terminates prematurely due to an error, no sampling is carried out. Conversely, if the optimization is successful, then all identified conformers are collected.

### 2.3 Validity and Accuracy Checks

At various stages of the active learning workflow, information is logged to allow for decision-making and tuning of the workflow. Specifically, information from *validity* and *accuracy checks* is used to decide when a system is to become inactive and determines the AL loop convergence. In this subsection, we discuss relevant settings and logged values/metrics for IR spectra.

*Validity* criteria are specific to each task. For the MD-*task*, we use the information about early stop conditions and then inspect the corresponding trajectories to see whether there are large energy changes between contiguous steps higher than 272 meV/atom.

For both the GO-*task* and the CF-*task* we check whether the structure optimizations converge, according to the ″Good″ settings of the AMS software, which are specified in Section 2.1.2.

As the GO-*task* in this paper delivers the Hessian as well, we can check for imaginary frequencies that indicate convergence to a saddle point on the PES. The obtained Hessian for each MLIP-EFD model is furthermore used to compute an IR spectrum that can be compared with the committee spectrum, obtained by diagonalization of the committee-averaged Hessian matrix. To be able to reduce this information to a scalar, we first need to compute the IR intensities on a predetermined frequency grid. For spectra obtained in the harmonic approximation, the intensity values at the chosen frequency grid {ω} are obtained via Lorentzian broadening:

3 $$A\left(\omega \right)=\overset{3N_{atoms}-6}{\underset{i=1}{\sum }}I_{i}\frac{1}{\pi }\frac{a}{{\left(\omega -F_{i}\right)}^{2}+a^{2}}$$

where *F*_*i*_ and *I*_*i*_ are, respectively, the harmonic frequencies and intensities, and *a* is a suitable broadening parameter, chosen as 30 cm^–1^ in this work. Given these discretized IR intensity vectors (*A* and *B*), we can then define the cosine similarity score:

4 $$\mathrm{CosIR}=\frac{\mathrm{A⃗}\cdot \mathrm{B⃗}}{∥\mathrm{A⃗}∥∥\mathrm{B⃗}∥}$$

which gives values in the range (−1, 1) with 1 signaling perfect agreement. As this value can be computed for each MLIP-EFD relative to the committee spectrum, we average these values once more to obtain the *Committee Agreement CosIR*. The target value is set to 0.9. As an illustration of its use, we report in Figure 3 in the top panel, the values of *Committee Agreement CosIR* during the AL iterations. This similarity score is logged by the GO-*task* during the IR calculation. As displayed in the bottom panel, in each AL iteration, the number of remaining active molecules decreases, reducing also the number of data points in the top panel. The effect of retraining the cMLIP is clearly visible, with the agreement between the committee increasing to a satisfactory value.

**Figure 3:**
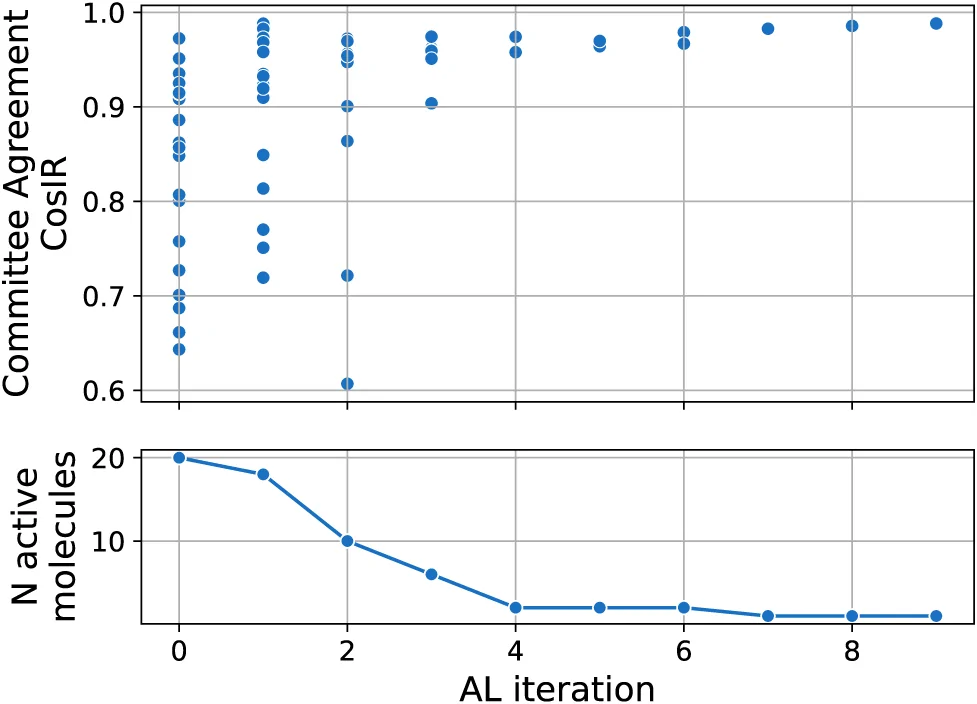
Top: the committee agreement based on the cosine IR values logged during an AL loop by the validity checker for the GO-*task*. Each point represents a molecule in the active systems set. Bottom: the number *N* of active molecules at each AL iteration. The data come from the AL loop identified as *als-*MT.

During the *accuracy checks*, metrics on newly added data are collected by comparing the properties obtained with cMLIP-EFD to those obtained with the reference method, in our case, DFT. The *accuracy checks* targets, shown in Table 1, are used to decide whether a system is already well described by the cMLIP-EFD and can be made inactive. In Figure 4 we report the mean absolute error (MAE) for energy, forces, and dipole moment. At each iteration, the metrics are evaluated on different structures and possibly different molecules as well. Therefore, similarly as seen in Figure 3, the decreasing errors show the ability of the retrained model to reproduce the DFT results. In this case, the error values decrease somewhat slower as more challenging frames get selected during the AL loop to capture information that was not yet present in the training set. Overall, Figure 4 shows the expected decreasing error trend, while incidental increases reflect iteration-to-iteration variability because the accuracy checks evaluate different adaptively sampled structures at each iteration.

**Table 1:** Summary of Target Metrics Used as Accuracy Criteria^a^

Property	Units	Metric	Target
Energy	meV/atom	MAE	7
Energy	meV/atom	RMSE	8
Forces	meV/Å	MAE	60
Forces	meV/Å	RMSE	90
Forces	meV/Å	MAE	90^b^
Dipole	mDebye	MAE	190
Dipole	mDebye	RMSE	240

a MAE and RMSE denote the mean absolute error and root mean square error , respectively.

b Per-atom type: the metric is evaluated separately for each atomic number.

**Figure 4:**
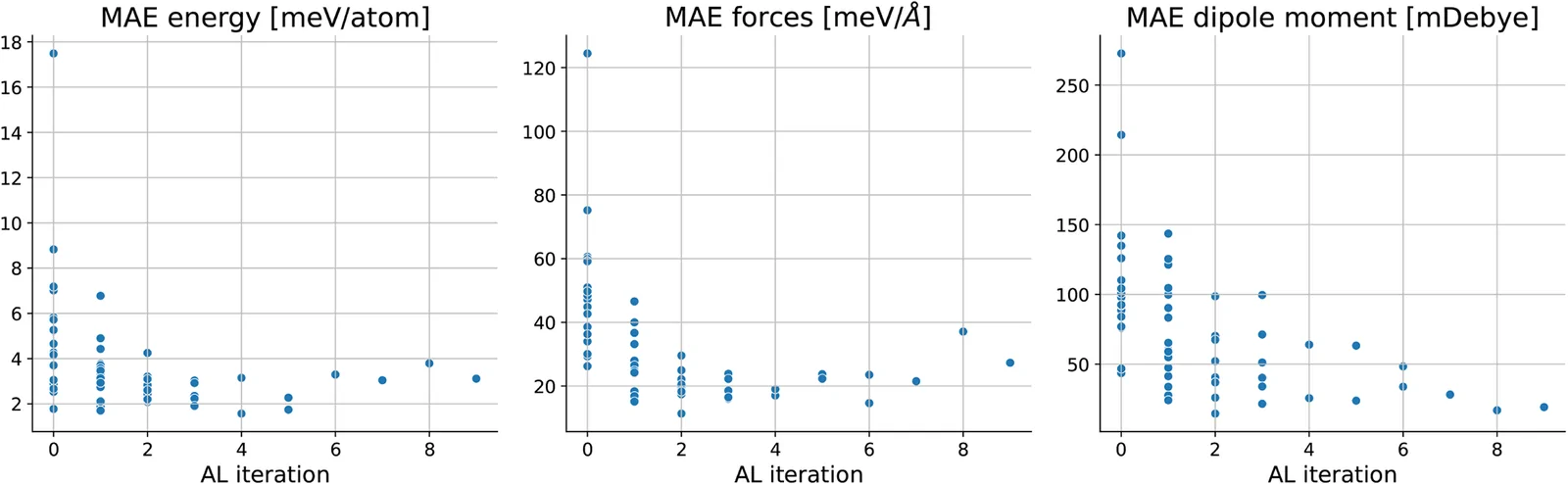
Mean absolute errors (MAE) collected during the *accuracy checking*. Each data point represents the MAE for an active molecule. The data come from the AL loop identified as *als-*MT.

Table 2 gives the error metrics obtained after the training of cMLIP-EFD is completed. These results are in line with error metrics obtained in earlier work.^35,36^ Furthermore, these values can be compared with the values collected by the *accuracy checks* reported in Figure 4 to see the effect of training. The novel data are learned well by the cMLIP-EFD as is evident from the 2–10 fold decrease of the error after training.

**Table 2:** Accuracy of the Final cMLIP-EFD Model on Training, Validation Sets for Properties: Energy (meV/atom), Forces (meV/Å), and Dipole (mDebye)^a^

Data set	Metric	Energy	Forces	Dipole
Training	MAE	1.8	4.6	17.1
	RMSE	2.5	6.0	23.9
	Entries	1132	66195	3396
Validation	MAE	2.0	13.8	30.9
	RMSE	3.0	25.2	50.1
	Entries	244	14217	732

a Entries denote the number of scalar reference values used for each property: structures for energy, Cartesian force components for forces, and Cartesian dipole components for dipole. The data come from the AL loop identified as *Als-*MT.

### 2.4 MLIP and Committee Architecture, Initialization, and Training

The selection of the MLIP architecture is of course crucial. Prior work has shown that using a combination of two models, one for energies and forces and another dedicated to dipole prediction (MLIP-EF+D), give accurate results.^21–24^ But MLIP-EF+D implies the disadvantages of double training/evaluation, implying extra computational overhead for the IR prediction. Some models are capable of predicting energy, forces, and electric dipole at the same time,^18,33,47–50^ and among these, we selected the MACE equivariant architecture.^33^ The model architecture is based on the class ″EnergyDipolesMACE″ for H, C, N, O, and S, with maximum equivariance order *l*_max_ = 3, two interaction layers, 64 channels, and cutoff radius of 4.0 Å. The total number of model parameters was 300 thousand, much lower than common universal MLIPs with around 10 million parameters.^51^ We used the MACE package (version 0.3.6).^33^ Training used a batch size of 5, a maximum of 2000 epochs, and the ″energy_forces_dipole″ loss function with energy, force, and dipole weights of 100 eV^–1^, 5 (eV/Å)^−1^, 1 (e Å)^−1^, respectively. The full settings in yaml format are reported in the Supporting Information. The training was performed on a single NVIDIA GeForce RTX 4090.

The cMLIP-EFD is composed of three models that were initialized with different random seeds and trained on an initial training and validation data set of 200 frames. These frames were generated by running MD-*task* at 1000 K, sampling five evenly spaced frames, and the GO-*task* task with normal mode sampling at 1000 K. The use of 1000 K is a system-specific choice intended to generate a broad, low-cost bootstrap data set. For larger and more flexible systems, lower or multiple temperatures or alternative initialization strategies may be more appropriate to avoid creating unrealistically distorted structures. Both tasks are run with the density functional-based tight-binding method (DFTB) with the parametrization GFN1-xTB.^14^ All other settings for the MD-*task* and GO-*task* are kept fixed, as specified in Section 2.2. Six frames were sampled for each task and molecule for a total of 240 frames (2 × 6 × 20 = 240). These structures were split by a stratified random training/validation split by drawing one frame per task and molecule, yielding 200 training and 40 validation structures. This initial cMLIP-EFD is considered a computationally inexpensive baseline.

During active learning, all committee members are retrained.

### 2.5 Reference Level of Theory

The reference level of theory chosen is DFT, utilized during the *frame-labeling* step of the active-learning loop as well as for testing the trained cMLIP-EFD. Simulations were performed using the Amsterdam Density Functional (ADF^52^) from the Amsterdam Modeling Suite (AMS^37^). We selected the XLYP GGA functional^53^ with double-ζ basis set. We chose this particular combination of exchange-correlation density functional approximation (DFA) and basis set to balance accuracy and computational efficiency. While we focus on accurately training the MLIP-EFD at this level of theory, our conclusions are not specific for the particular DFT functional approximation (DFA) used, because MLIPs have an inherent flexibility, which makes them also suitable for other levels of theory. In selecting the DFA for this work, we decided to focus on the short range and not include dispersion corrections. These additional dispersion components of most modern DFAs are designed to treat long-range effects that are rather independent of the particular choice of semilocal DFA.

### 2.6 Test Sets

We prepared four test sets. We note that while the molecule sets used in testing coincide partially with those used in the AL loop, the AL loop is only provided with initial structures and therefore has no knowledge of DFT-optimized structures or of DFT-computed spectra.

The test sets are (i) *test-*20AA, consisting of DFT-calculated harmonic infrared spectra of 20 amino acids in their nonzwitterionic state for a single conformer per amino acid (reported in Figure 5). The settings used for geometry optimization and frequency calculation are identical to those described in Subsection [Sec sec2.2]. (ii) *test-*20AAC, which has the same molecules as *test-*20AA but has their IR spectra computed differently, as a Boltzmann-weighted average (at 298 K) from low-energy conformer spectra. The Boltzmann-weighted IR spectrum was considered converged when the cumulative Boltzmann population exceeded 99%. For the conformer search, we used the AMS^37^ implementation with the RDKit^46^ conformer search. The workflow for the DFT reference data consists of ″generate″ and ″optimization″ tasks. The generate tasks used DFTB with the parametrization GFN1-xTB^14^ and were followed by the ″optimization″ task with DFT and a final single-point calculation within the double harmonic IR approximation with the settings as reported in Subsection [Sec sec2.2]. (iii) *test-*10DP, spectra obtained as in *test-*20AA but for 10 dipeptide molecules (shown in Figure 5, with two-letter abbreviations), and (iv) *test-*7AAMD, seven IR spectra computed for alanine, cysteine, glycine, proline, serine, threonine, and valine amino acid molecules. They are obtained with DFT-based molecular dynamics at 300 K, thus including anharmonic effects. The DFT-based molecular dynamics calculations, used only for generating reference IR spectra of each molecule, consisted of 3 stages: NVT equilibration phase for 5 ps simulation at 300 K (with Nosé–Hoover thermostat, damping coefficient of 200 fs, time step 1 fs), then 10 ps NVT production phase at 300 K (with Nosé–Hoover thermostat, damping coefficient 200 fs, time step 1 fs) to sample three starting conditions, and last 200 ps constant-particle-number, volume, and energy (NVE) simulations (time step 0.5 fs). The reference IR spectra are then derived from the dipole moment time series evaluating the Fourier transform of the autocorrelation function of the dipole moment time derivative:

5 $$A_{\omega }\propto \int {\langle \mathrm{\mu ̇}(\tau )|\mathrm{\mu ̇}(t+\tau )\rangle }_{\tau }e^{-i\omega t}dt$$

**Figure 5:**
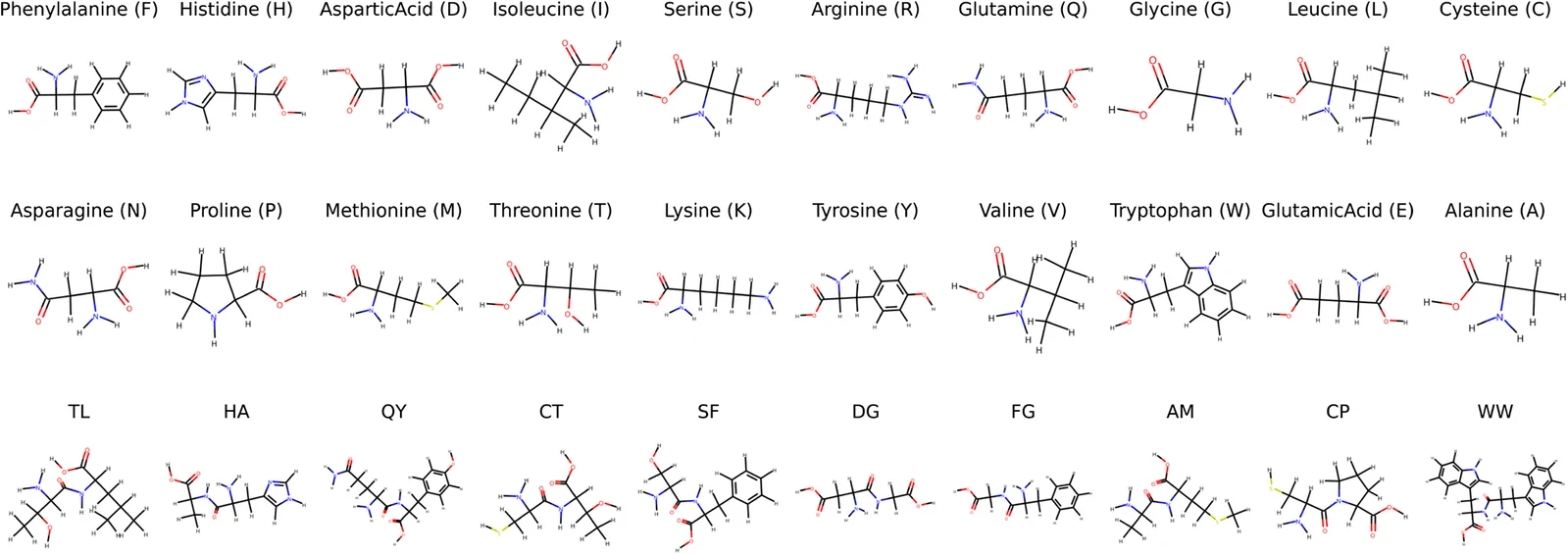
Molecules considered in this study. Top two rows: amino acids with their corresponding single-letter abbreviations. Bottom row: dipeptides.

We used the implementation in SchNetPack^54^ for calculating the Fourier transform of the autocorrelation function of the dipole moment time derivative. The final IR spectrum used to test the adequacy on our learning scheme is the average of these three runs.

The test sets are used to compare the predicted IR spectra, in terms of frequencies and intensities, to similarly calculated DFT reference spectra. This goes beyond the training and validation sets constructed during the active learning loop in several ways: DFT IR spectra are never computed during AL; harmonic IR spectra computed at the cMLIP-EFD level during AL were only used for measuring the uncertainty within the committee, the test sets explore more local minima (*test*-20AAC) and larger structures (*test*-10DP), and anharmonic effects are probed by using a different method to generate the IR spectrum (*test*-GlyMD).

### 2.7 Test Phase: Infrared Spectra Calculation

After completion of the AL loop, we have a cMLIP-EFD that is to be used in production runs, to efficiently substitute DFT at a comparable accuracy. To test whether our AL method can reach this goal, we generate cMLIP-EFD IR spectra and then compare these with the data sets obtained at the DFT level, as presented in Subsection [Sec sec2.6]. We will consider the similarity as well as the dissimilarity between the spectra to better understand how errors can impact compound determination in production workflows. For *test*-20AA, we also look in more detail at how well normal modes are reproduced.

#### 2.7.1 Spectra Prediction Errors

We first consider the reproduction of harmonic IR spectra using the CosIR score as defined in Equation 4. For *test-*20AA and *test-*10DP the settings are thereby identical to the ones described in subsection [Sec sec2.2], but the initial geometry is now taken from the test set to make sure we compare the same conformers. For the comparison of Boltzmann-weighted IR spectra in *test-*20AAC, the cMLIP-EFD procedure used consisted of just the ″generate″ task, followed by single-point double-harmonic IR calculation. The Boltzmann-weighted IR spectra were then computed using a temperature of 300 K (spectra at other temperatures are listed in the Supporting Information; they show that the temperature dependence is quite small for these molecules) and compared to the reference IR spectra of the test set. Finally, to perform the comparison for *test-*GlyMD, we run only the NVE simulations with the cMLIP-EFD.

#### 2.7.2 Spectra Similarity Assignment

Besides faithfully reproducing reference spectra for a given molecule, it is also of interest to be able to identify molecules on the basis of their spectrum. To check whether this goal can be reached, we assess spectral agreement between the cMLIP-EFD and DFT-predicted spectra and check whether we can correctly pair spectra from the same molecules within the set of investigated molecules. To this end, we implemented a spectral assignment algorithm to establish pairwise correspondences between the cMLIP-predicted spectra and those from DFT. The procedure builds a cost matrix using CosIR and then performs the assignment with the Hungarian algorithm.^55^ By solving the full assignment problem, the Hungarian algorithm automatically returns the set of spectral pairs that minimize the overall CosIR-based cost. Although other, potentially better, metrics could be used, CosIR was chosen for consistency with previous sections.

#### 2.7.3 Normal Modes Comparison

When comparing two IR spectra obtained within the harmonic approximation, it is possible to use the detailed normal mode information that is suppressed in CosIR comparisons. To do so, we implemented two algorithms to assess the similarity of normal modes in computed spectra.

i The first and simplest approach is inspired by the calculation of Franck–Condon factors,^56^ in which one directly compares normal-mode vectors by calculation of the Duschinsky matrix (*D*). As shown in Figure 6 for a representative example, the Duschinsky matrix allows for a quick visual inspection of the similarity. In this case, diagonal elements are clearly dominant and close to 1, indicating high similarity of the normal modes of the two spectra. By performing the Hungarian algorithm on this matrix (with opposite sign), we find in a few cases that swapping modes yields a better similarity, these are therefore reassigned (tagged with red arrow and ″A″ in Figure 6). This is particularly important for dense regions in the spectra where a simple assignment based on sorting of eigenvalues can easily be incorrect. After this possible mode swapping, we collect two quantities that indicate the similarity of the normal modes: the mean Duschinsky matrix diagonal elements (⟨diag­(D)⟩) and the Duschinsky matrix mean assignment cost (⟨Cost_D_⟩). Given that ⟨diag­(D)⟩ ≤ ⟨Cost_D_⟩, the distance between these two values is informative for understanding how the diagonal terms predominate in the Duschinsky matrix. For the example in Figure 6 these two values are ⟨*tr*(D)⟩ = 0.92 and ⟨Cost_D_⟩ = 0.96.
ii For a more robust comparison that allows for mode mixing in dense regions of the spectrum, we utilize the partial vibrational density of states (PVDOS). The PVDOS, for atom *I* and normal mode *n*, is defined as
6 $$P_{I,n}=\frac{m_{I}{|{\mathrm{\eta ⃗}}_{I,n}|}^{2}}{\underset{J}{\sum }m_{J}{|{\mathrm{\eta ⃗}}_{J,n}|}^{2}}$$
  Here, *m*_*I*_ represents the atomic mass of atom *I*, and *η⃗*_*I*,*n*_ denotes the displacement vector for atom *I* in the normal mode *n*. According to its definition, this quantity is invariant by rotations and translations. To address the permutational invariance of atoms, we sum across each atom type, distinguishing hydrogen atoms based on the atom to which they are bonded to. As a result, we get the quantity: *P_Z̃,n_* corresponding to the atom type *Z̃* that is no longer influenced by rotations and atom permutations. Ultimately, this allows us to derive the relative intensities for each mode: *P_Z̃,n_A_n_= Ã_Z̃,n_*. The spectrum can be decomposed in atom-type contributions (see Figure 7). Each atomic contribution to the IR spectrum can be compared with the reference using the cosine similarity score, we refer to it as $\left\langle {\mathrm{CosIR}}_{\mathrm{PVDOS}}^{\mathrm{byAtom}}\right\rangle$ .

**Figure 6:**
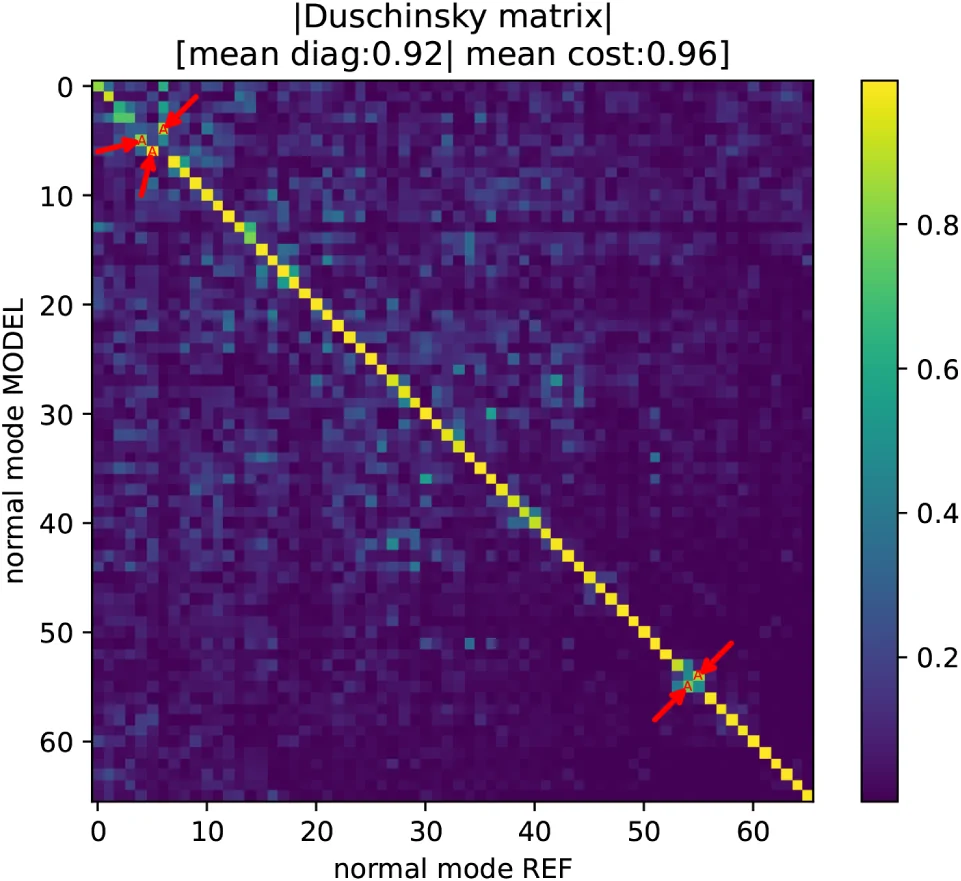
Duschinsky matrix absolute values, comparing normal modes of lysine amino acid for *als-*MT cMLIP-EFD and reference DFT. We highlight the off-diagonal modes assigned based on Hungarian algorithm with a red A and arrow.

**Figure 7:**
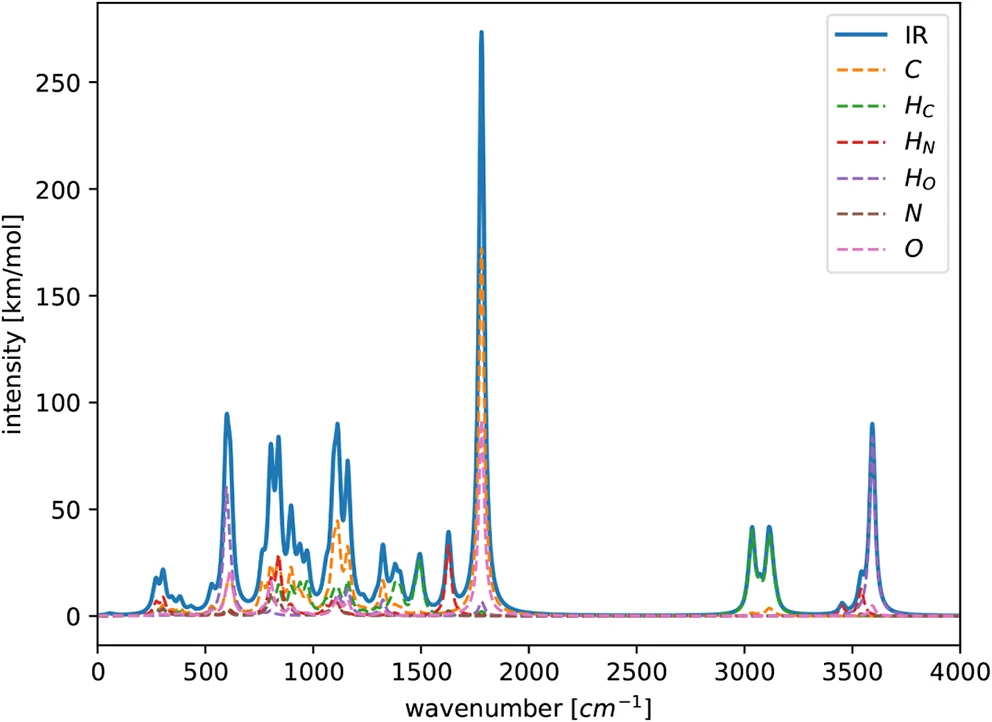
Infrared (IR) spectra of valine and its decomposition into atomic contributions via the partial vibrational density of states. Hydrogen atoms are differentiated based on the atoms they are bonded to, such that *H_O_* denotes a hydrogen atom bonded to an oxygen atom.

## 3 Results and Discussion

### 3.1 Robust Active Learning Strategies

We compare two distinct active learning strategies (*als*-) in terms of the robustness of the final model: *als*-ST and *als*-MT. *als-*ST is a single-task (ST) strategy with only the GO-*task*. *als-*MT is a Multi-Task (MT) strategy and includes all the tasks in Figure 2: GO-*task*, MD-*task*, and CF-*task*. We ran these AL workflows three times each.

#### 3.1.1 IR Prediction of Glycine across Methodologies

We report in Figure 8 the spectra predictions for *als-*MT (run #1) and *als-*ST (run #1) on the glycine molecule on the different data sets. This example illustrates that *als-*MT exhibits superior accuracy in predicting IR spectra compared to *als-*ST, using three different methodologies. On *test-*GlyMD, *als-*ST shows similar CosIR as for *als-*MT (0.92 and 0.94 respectively), which indicates that the harmonic exploration of the PES yields structures sufficiently representative of those encountered in the molecular dynamics trajectory at 300 K. At closer inspection, it becomes apparent that an important peak around 1400 cm^–1^ is absent in the *als-*ST spectrum and present in *als-*MT. This illustrates how sampling directly for the data distribution under examination is important. This difference in spectral prediction is reflected by the CosIR, which shows a value difference of 0.02, indicating the order of magnitude that should be considered to adequately penalize the absence of chemically relevant peaks. Near 3500 cm^–1^, *als-*MT reproduces both OH-stretch peaks but overestimates the intensity of one; this may stem from dipole-derivative errors and/or force-field inaccuracies affecting broadening and conformer populations. When examining the *test-*20AAC top trace, the *als-*ST reconstruction fails to recover two relevant bands at 1400 and 3100 cm^–1^, which are correctly identified by *als-*MT. This difference in accuracy is reflected in the CosIR values, which are 0.89 for *als-*ST and 0.92 for *als-*MT (a difference of 0.03). From Figure 8 the limitations of CosIR are indeed evident and, for spotting chemically relevant differences, it is recommended to also plot and visually compare the spectra. Being a global score, CosIR emphasizes overall agreement rather than local discrepancies. The major advantage of CosIR is its simple definition, making it easy to predict its behavior and limitations. In the Supporting Information, we provide a comparison with other similarity measures, following the approach of Esch et al.^57^

**Figure 8:**
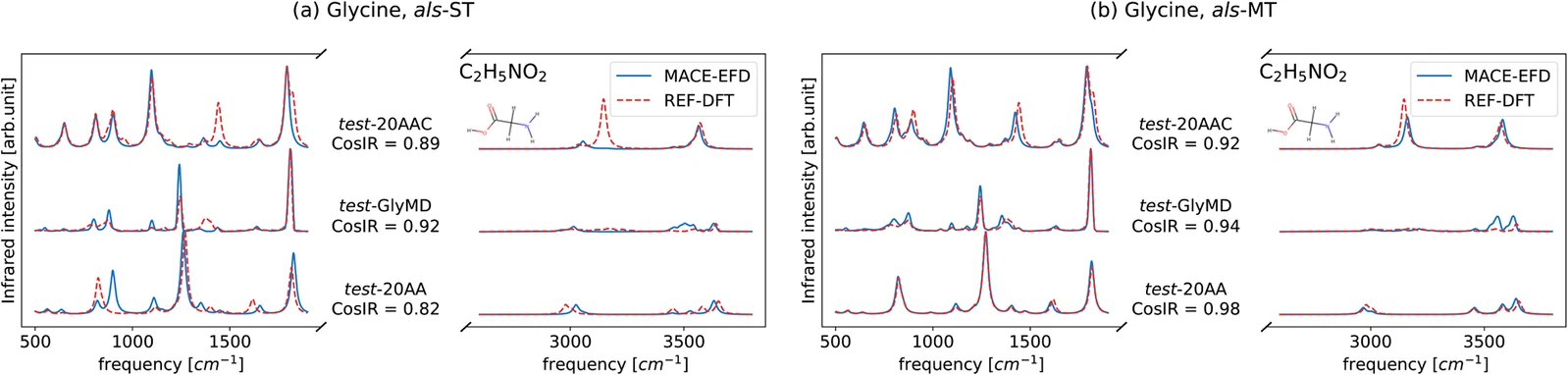
DFT and MLIP-EFD IR spectra predicted for the glycine molecule using three different approaches, corresponding to the test sets *test-*20AA, *test-*GlyMD, and *test-*20AAC. In the panels, the MLIP-EFD results shown are those obtained from (a) *als-*ST and (b) *als-*MT.

To complement the spectral-accuracy analysis, we also compare the computational cost of the three workflows for a node comprising 24 CPU cores and 1 GPU, which is representative for current-day hardware. For glycine, the wall-clock times indicate an approximately 6-fold speed-up for GO and a 29-fold speed-up for MD, whereas the current conformer implementation was slightly (1.6×) slower, primarily because the multiple geometry optimizations do benefit from CPU parallelization but not from the GPU parallelization that makes ML workflows efficient. Given these hardware specifics, these timings should be regarded as indicative rather than definitive, as our current workflows are not yet specifically optimized for MLIP-EFD calculations and may not represent the performance achievable with a fully optimized implementation.

#### 3.1.2 IR Prediction on *Test-*20AAC

We further inspect the full *test-*20AAC results in Figure 9 for run #1. In the Supporting Information, we report the results of the remaining runs. The *als-*ST model fails to predict the peaks in the regions around 1400 cm^–1^ and above 2800 cm^–1^ (in gray color, Figure 9a). These peaks arise for conformations with intramolecular hydrogen bonding. The *als-*MT approach performs much better (Figure 9b), with most of the peaks being well described and the CosIR values being significantly higher than for *als-*ST. A few molecule-specific cases in Figure 9, namely methionine, leucine, glutamic acid, and asparagine, show higher CosIR for *als-*ST than for *als-*MT in run #1; we attribute these exceptions to the low conformational coverage in the AL CF-getter sampling. Methionine and asparagine show small or run-dependent differences, whereas leucine and glutamic acid are likely more sensitive to sampled side-chain rotamers and hydrogen-bonding motifs. However, for arginine, asparagine, aspartic acid and histidine, the peaks between 2700 and 3000 cm^–1^ are still missing. We attribute this error to an incomplete coverage of the AL conformational space. A possible solution would be to expand the CF-*task* during the AL to collect a larger number of conformers for flexible molecules.

**Figure 9:**
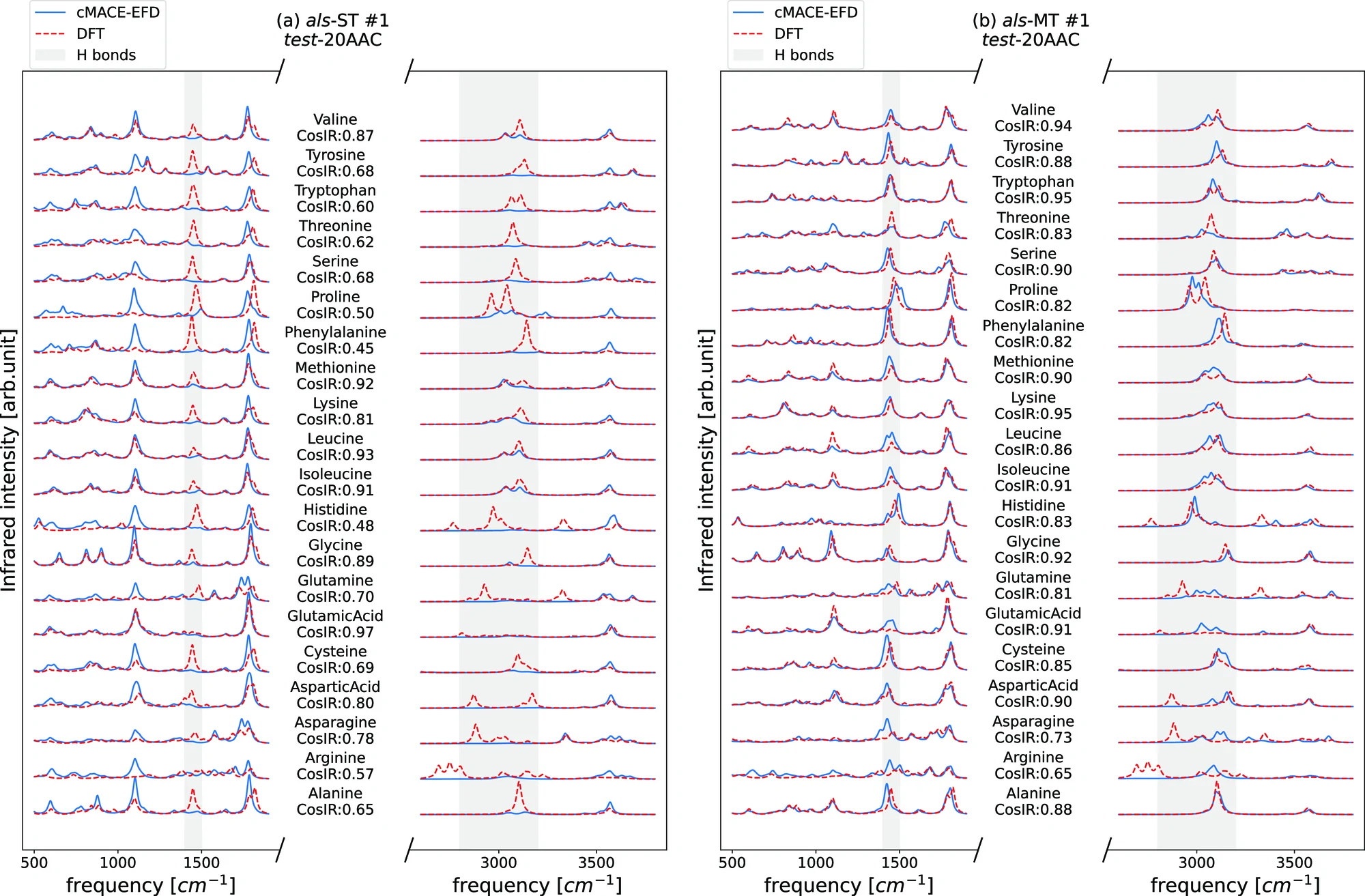
Boltzmann-averaged infrared spectra at 298 K from the *test-*20AAC test set: (a) *als-*ST, (b) *als-*MT.

#### 3.1.3 IR Prediction Across Test Sets

We can now look at the average results on the CosIR across the different test sets and runs in Table 3. In this table, we also report the number of iterations the active learning loop took to converge $N_{\mathrm{iter}}^{\mathrm{AL}}$ , the final training data set size (*N*_train_), and the total time taken for the AL to converge (*t*_AL_). The baseline model is the starting model used for all the AL workflow, prepared as explained in subsection [Sec sec2.4].

**Table 3:** Robust Active Learning Strategies^a^

AL strategy	AL run	$N_{\mathrm{iter}}^{\mathrm{AL}}$	*N*_train_	*t*_AL_	⟨CosIR⟩ *test-*20AA	⟨CosIR⟩ *test-*7AAMD	⟨CosIR⟩ *test-*20AAC	⟨CosIR⟩ *test-*10DP
Baseline	#1	0	200	3 h	0.81	0.87	0.74	0.72
*als-*MT	#1	9	1132	50 h	0.94	0.92	0.86	0.74
*als-*MT	#2	3	929	24 h	0.94	0.93	0.87	0.74
*als-*MT	#3	9	1046	52 h	0.92	0.95	0.88	-
*als-*ST	#1	5	893	33 h	0.91	0.92	0.72	0.74
*als-*ST	#2	3	817	27 h	0.91	0.92	0.71	0.72
*als-*ST	#3	2	817	19 h	0.91	0.91	0.74	0.74
*als-*MT-AA|DP	#1	8	863	57 h	-	-	-	0.93

a We compare the final model obtained from different AL strategies: multi- or single-task (MT or ST). Most AL runs were done multiple times , with a corresponding column distinguishing them.

On *test-*20AA, both *als-*MT and *als-*ST provide a CosIR increase over the baseline, with improved accuracy for *als-*MT. For *test-*20AA, the testing procedure aligns with the one performed by Pracht et al.^23^ If we compare their results with ours, our final models have higher average accuracy (0.93 for *als-*MT and 0.91 for *als-*ST) than the one reported for their best performing model (0.86). Therefore, our AL strategy can be used to enhance the accuracy of the available MACE-EF+D models for specific systems. By examining the *test-*7AAMD column, we can derive analogous conclusions (for a detailed, spectrum-resolved analysis of the IR data for this test set, we refer to the Supporting Information). Notably, *als*-ST continues to accurately reproduce the IR spectra obtained from molecular dynamics (MD) simulations; however, *als*-MT still outperforms *als*-ST.

Moving from *test-*20AA to the more challenging *test-*20AAC, we find for all AL workflows, a decrease of CosIR. This decrease in accuracy is much smaller for the *als*-MT workflows than for *als*-ST, indicative of a more robust MLIP-EFD for challenging tasks. For *als*-ST, CosIR does not exhibit an increase relative to the baseline value; instead, the average CosIR decreases. This behavior may be interpreted as a manifestation of overfitting within the active learning framework, whereby the model becomes excessively specialized to specific regions of the PES.

Overall, *als-*MT systematically outperforms *als-*ST in all the test sets except *test-*10DP, where they have similar performance. Therefore, *als*-MT presents higher robustness on exploring a larger portion of the PES. This comes with the cost of a more computationally expensive workflow, taking longer to converge (average *t*_AL_ increases from 26 to 42 h) and more training data (average *N*_train_ increases from 800 to 1000). If we compare the baseline with *als-*ST, we see improvements on *test-*20AA but less so for *test-*20AAC. This gives an indication that including higher variety in the *tasks* is a simple and effective way to generate more robust MLIPs, i.e., capable of describing accurately larger portions of the PES.

On test set *test*-10DP, which concerns molecules unseen in the training, the *als*-ST and *als*-MT approaches give the same accuracy. This is mainly due to the undersampling of chemical environments in the training set around amide bonds, which is typical of dipeptides. We can improve the accuracy of the models on *test-*10DP by performing an active learning loop with dipeptides in the *active systems* set and by initializing MLIP-EFD with the model obtained as the outcome of *als-*MT run 1, rather than with the baseline model. This workflow is named *als-*MT-AA|DP, and the models obtained show an accuracy of the MLIP-EFD similar to that of the MLIP-EFD obtained at the end of the *als-*MT. The first warm-started *als-*MT-AA|DP checkpoint (with 182 training structures; 6.7 h) gives a mean CosIR of 0.80 on *test-*10DP, whereas the final checkpoint (863 structures; 57 h) reaches 0.93. Thus, the additional active learning iterations improve the mean CosIR by 0.13. This provides evidence for the transferability of the proposed approach to larger systems and demonstrates the incremental accuracy improvements achievable through successive iterations.

### 3.2 Use of cMLIP-EFD in IR Spectra Interpretation

In this section, we discuss how cMLIP-EFD can be used to aid in the interpretation of IR spectra. We apply the methodologies introduced in Subsection [Sec sec2.7]: molecular assignment from IR spectra based on spectra assignment, and peak assignment of an IR spectrum for a known compound/molecule by comparing normal modes.

#### 3.2.1 Similarity Between Spectra

As a first example, we take the data from *test-*20AAC and *test-*20AA, which each provide 20 reference spectra. These thereby function as surrogates for experimentally measured spectra. The same set of spectra is then also computed with cMLIP-EFD, and we now assume that the exact mapping between the spectra in the reference set and those computed by cMLIP-EFD is unknown. We then matched each cMLIP-EFD spectrum to the correct reference spectrum. In this way, cMLIP-EFD spectrum calculation acts as a classifier.

The results are reported for both *test-*20AAC and *test-*20AA in Table 4. Contrary to the previous findings in Table 3, the results indicate that models obtained as results from the *als-*ST exhibit superior spectra discrimination on the *test-*20AAC data set with respect to the models obtained from *als-*MT. This example highlights two concepts, first, improved similarity does not correlate with improved assignment reliability, second, a 0.9 CosIR accuracy could be a rule-of-thumb threshold to obtain good scores in the spectra assignment as seen in the results for *test-*20AA in Table 4.

**Table 4:** Similarity between Spectra. Misclassification Count of Spectra with Respect to the Total (20) for Both Test Sets: *Test-*20AAC and *Test-*20AA

		Misclassification count	
AL strategy	AL run	*test-*20AA	*test-*20AAC
Baseline	#1	5	5
*als-*MT	#1	0	6
*als-*MT	#2	0	4
*als-*MT	#3	0	4
*als-*ST	#1	0	0
*als-*ST	#2	0	0
*als-*ST	#3	0	4

#### 3.2.2 Normal Modes Comparison

The aim of the second example is to assess how accurately normal modes can be predicted for the known molecules. Knowledge of the nature of the normal modes is often crucial to the interpretation of spectra. There have been many documented efforts to improve the interpretation of spectra, both theoretically (for instance, by localizing normal modes to boost interpretability^58–60^ ) and experimentally^61^ (for example, through automatic peak assignment of experimental spectra,^62^ using machine learning techniques,^63^ or systematic workflows^64^).

In this subsection, we will focus on *test-*20AA set. For the purpose of normal mode classification, we first apply the methodologies introduced in Paragraph 2 in Subsection [Sec sec2.7]. By examining the average of the Duschinsky matrix diagonal values (⟨diag­(D)⟩) in Table 5, it is evident that *als-*MT provides a better description of the normal modes than *als-*ST. Also, in terms of final cost after assignment (⟨Cost_D_⟩) as reported in Table 5, *als-*MT performs best in this application. By comparing ⟨diag­(D)⟩ and ⟨Cost_D_⟩ we get to similar conclusions, but reporting these results using the Hungarian algorithm allows us to systematically exclude potential effects arising from incorrect mode assignment.

**Table 5:** Normal Modes Comparison.^a^

AL strategy	AL run	⟨diag(D)⟩	⟨Cost_D_⟩	$\left\langle {\mathrm{CosIR}}_{\mathrm{PVDOS}}^{\mathrm{byAtom}}\right\rangle$
Baseline	#1	0.75	0.87	0.75
*als-*MT	#1	0.90	0.94	0.93
*als-*MT	#2	0.92	0.96	0.92
*als-*MT	#3	0.88	0.93	0.89
*als-*ST	#1	0.85	0.91	0.88
*als-*ST	#2	0.88	0.93	0.89
*als-*ST	#3	0.87	0.92	0.90

a Overview statistics regarding the Duschinsky matrix (*D*): the mean of the diagonal elements (⟨diag­(D)⟩), mean assignment cost (⟨Cost_D_⟩) and the average cosine similarity on the PVDOS weighted spectra by atom $\left\langle {\mathrm{CosIR}}_{\mathrm{PVDOS}}^{\mathrm{byAtom}}\right\rangle$ . The results are applied to the *Test-*20AA test set.

An alternative method to compare normal modes is via the PVDOS, using the $\left\langle {\mathrm{CosIR}}_{\mathrm{PVDOS}}^{\mathrm{byAtom}}\right\rangle$ score introduced in section 2.7.3. The results in Table 5 in the corresponding column clearly show that poor performance on the CosIR (in Table 3) is also apparent in the $\left\langle {\mathrm{CosIR}}_{\mathrm{PVDOS}}^{\mathrm{byAtom}}\right\rangle$ score.

The lowest performance observed in the $\left\langle {\mathrm{CosIR}}_{\mathrm{PVDOS}}^{\mathrm{byAtom}}\right\rangle$ for *als-*ST models is associated with hydrogen atoms bonded to oxygen, exhibiting a cosine similarity of 0.65 (see Table S4). In contrast, *als-*MT demonstrate significantly higher performance metrics, exceeding a cosine similarity of 0.71. Analyzing the distribution of atom types across the 20 amino acids reveals that the least abundant atom types are nitrogen (N), hydrogen bonded to oxygen (*H*_*O*_), sulfur (S), and hydrogen bonded to sulfur (*H*_*S*_), with respective counts of 29, 25, 2, and 1 (against 120 occurrences for hydrogens bonded to carbon *H*_*C*_). Consequently, it is not surprising that these atom types present challenges in accurately learning their IR-PVDOS-weighted contributions. On the other hand, multitask active learning models result in greater improvement in interpretability.

#### 3.2.3 Experimental IR Spectra Comparison

Figure 10 compares digitized gas-phase FTIR spectra for ten amino acids reported by Linder et al.^65,66^ with conformer-averaged XLYP/DZP and *als-*MT run #1 cMLIP-EFD spectra. The cMLIP-EFD curves closely follow the DFT reference, while the differences between the experimental and DFT are generally larger.

**Figure 10:**
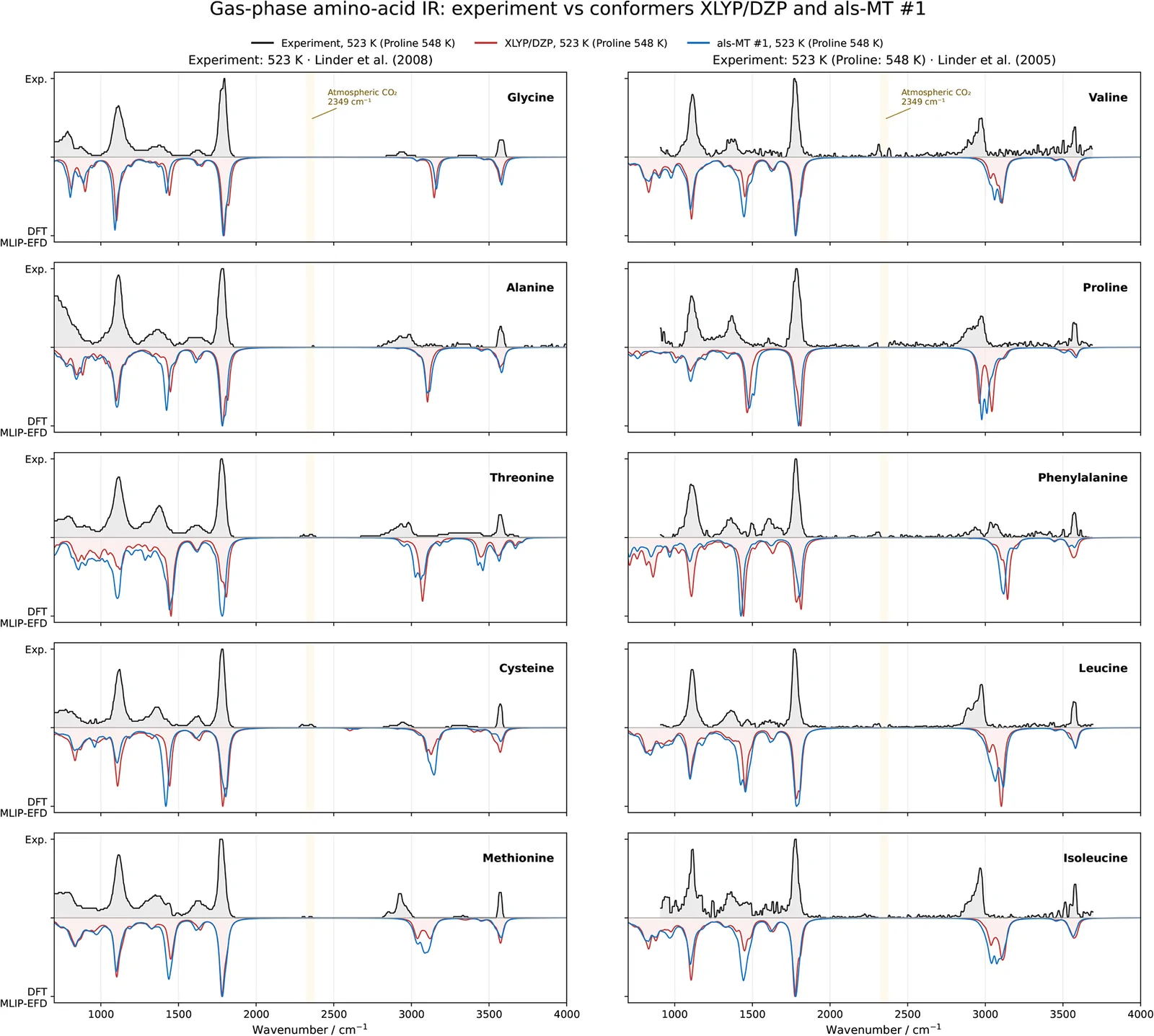
Digitized experimental gas-phase FTIR spectra (black, upward) reported by Linder et al.,^65,66^ compared with conformer-averaged harmonic spectra obtained with XLYP/DZP (red, downward) and the *als-*MT run #1 cMLIP-EFD (blue, downward). Conformer populations were evaluated at the measurement temperature (523 K, except 548 K for proline); the calculated spectra use 30 cm^–1^ broadening and unscaled frequencies, and each trace is independently peak-normalized. The shaded region at 2349 cm^–1^ marks residual atmospheric CO_2_ in the experimental spectra.

This comparison should therefore not be used to judge the intrinsic quality of the cMLIP-EFD, which was trained to reproduce XLYP/DZP rather than experiment. Instead, it indicates that the cMLIP-EFD has reached an accuracy level at which the experiment–DFT discrepancy exceeds the DFT–cMLIP-EFD discrepancy. The main differences of cMLIP-EFD-generated spectra with respect to experiment therefore arise from other issues.

### 3.3 Efficient Active Learning Strategies

Given that *als-*MT is a more robust option, we investigated whether there are straightforward approaches to increase data efficiency and reduce computational time, without sacrificing accuracy. To this end, we conducted an additional batch of experiments with two slightly different active learning strategies: *als-*MT-filter and *als-*MT-filter-stop. The *als-*MT-filter strategy uses the same settings as *als-*MT but adds an extra *prefilters* step to remove duplicates from the *candidate frames*. Duplicate removal is performed by aligning candidate structures with the Kabsch algorithm, computing atom-order-preserving root-mean-square deviation values, and applying complete-linkage agglomerative clustering with a distance threshold of 0.05 Å; the full workflow is described in the Supporting Information. This variant of *als-*MT is referred to as *als-*MT-filter. Including a filter of this kind in our workflow is justified because different *frame getters* operate independently and may generate or sample identical structures. The *als-*MT-filter-stop is analogous to *als-*MT-filter with an added early stopping callback. This callback declares the active learning loop converged when fewer than two *active systems* remain. This is beneficial because, after the active set has been reduced to only a few molecules, further additional iterations typically yield only marginal improvements while consuming disproportionate computational resources.

In Table 6 we report the $N_{\mathrm{iter}}^{\mathrm{AL}}$ , *N*_train_ and *t*_AL_ as well as CosIR on the *test-*20AAC test set. We use this table to compare *als-*MT, *als-*MT-filter and *als-*MT-filter-stop in terms of accuracy, data, and time efficiency. To do so, we build a loss given by Loss = 0.01*N*_train_ + 0.08*t*_AL_ + 100­(1 – ⟨CosIR⟩^test–20AAC^). The normalization coefficients are set such that a change in the value can be considered as significant. The corresponding Loss values are also reported in Table 6. From that loss, we highlight how *als-*MT-filter-stop is the most data-efficient method. It keeps similar accuracy of *als-*MT while becoming closer in *t*_AL_ with the *als-*ST results reported in Table 3 (27 h vs 23 h as median values for *t*_AL_). However, early stopping may not always constitute the most effective strategy, as it can penalize accuracy improvements for more challenging systems. In particular, we observed a shift of the lowest 25% of values toward smaller CosIR values for *test-*20AAC, with a corresponding change in CosIR of approximately 0.1. This observation suggests that, in future work, it would be valuable to develop a Frame Getter that adaptively increases the number of samples as a function of validity and accuracy assessments. In such a framework, data would be sampled more extensively at earlier stages of the process, with the aim of reducing the total number of iterations required to achieve the desired performance. Finally, *als-*MT-filter reduces *N*_train_ but does not substantially improve *t*_AL_. Adding the early stopping callback greatly reduces *t*_AL_ and therefore yields lower loss values. These results suggest that similar intuition on machine learning training procedures could be transferred to on-the-fly active learning workflows.

**Table 6:** Data Efficiency^a^

AL strategy	AL run	$N_{\mathrm{iter}}^{\mathrm{AL}}$	*N*_train_	*t*_AL_	⟨CosIR⟩	Loss
*als-*MT	#1	9	1132	50 h	0.86	29
*als-*MT	#2	3	929	24 h	0.87	24
*als-*MT	#3	9	1046	52 h	0.88	27
*als-*MT-filter	#1	5	877	29 h	0.85	26
*als-*MT-filter	#2	9	1097	45 h	0.86	29
*als-*MT-filter	#3	5	991	31 h	0.85	27
*als-*MT-filter-stop	#1	3	826	23 h	0.84	26
*als-*MT-filter-stop	#2	3	782	22 h	0.89	21
*als-*MT-filter-stop	#3	5	1010	31 h	0.90	23

a We compare the final model obtained from different AL strategies: *Als-*MT, *Als-*MT-Filter and *Als-*MT-Filter-Stop. Most AL runs were done multiple times , with a corresponding column distinguishing them . The ⟨CosIR⟩ refers to the test set *Test-*20AAC. The loss (lower is better) is the sum of the weighted contributions of *N*_train_ and *t*_AL_ as well as CosIR on the *Test-*20AAC test set: Loss = 0.01*N*_train_ + 0.08*t*_AL_ + 100­(1 – ⟨CosIR⟩^test–20AAC^).

## 4 Conclusion

In this work, we developed a flexible on-the-fly active learning workflow for modeling IR spectra. By combining geometry optimization, molecular dynamics, and conformer search tasks within a multitask framework, the workflow efficiently generated training data and produced models that were more robust than the single-task alternative across harmonic, conformer-averaged, and dynamical IR tests. The multitask strategy consistently provided a better description of IR spectral line shapes, especially for challenging cases involving conformational diversity and intramolecular hydrogen bonding. At the same time, the spectral assignment study showed that improved spectral prediction does not necessarily translate to better spectral discrimination between molecules. For this reason, metrics such as CosIR should be complemented by assignment-based tests when the final goal is molecular identification.

We also showed that the introduction of frame filtering and early stopping can reduce the computational cost with only a minor loss in accuracy. Overall, this study demonstrates that our on-the-fly active learning approach is a practical and data-efficient route for building system-specific machine-learned potentials for vibrational spectroscopy, with clear potential for extension to more complex workflows in which robust MLIP-EFD models are required. A central strength of the workflow is its modular design, which allows straightforward extension with additional tasks and supports the treatment of sets of target molecular species within the same active-learning framework.

In future work, we plan to use newly developed universal MLIPs as starting points for our active learning workflows. We also aim to apply the methodology to larger and more diverse molecular sets, while further expanding the *tasks* and *frame getters* components to improve the coverage of conformational space.

The results presented here were obtained using a single NVIDIA GeForce RTX 4090, MACE models with about 300 thousand parameters, and relatively small training data sets of around 1000 samples. In this sense, the present work contributes to the development of active-learning workflows capable of deploying smaller MLIPs on more affordable GPU architectures. While current trends in AI often favor larger models and data sets to maximize accuracy, this work highlights the value of data efficiency and compact models.

## Supplementary Material



## Notes

Active-learning workflow schematic; computational settings for DFT calculations, molecular dynamics, geometry optimizations, conformer searches, and MACE training; duplicate-removal workflow; comparison of configurational-space coverage across independent multitask active-learning runs; energy–force comparison of newly acquired frames; reference infrared spectra for the amino acid test sets; conformer-averaged and molecular-dynamics-based infrared spectra across independent active-learning runs; atom-resolved partial-vibrational-density-of-states similarities; comparison of spectral similarity measures; temperature-dependent Boltzmann-weighted infrared spectra (PDF)

## References

[1] Popa D., Udrea F. (2019). Towards Integrated
Mid-Infrared Gas Sensors. Sensors.

[2] Willer U., Saraji M., Khorsandi A., Geiser P., Schade W. (2006). Near- and
Mid-Infrared Laser Monitoring of Industrial Processes, Environment
and Security Applications. Opt. Lasers Eng..

[3] Leung
Tang P., Alqassim M., Nic Daéid N., Berlouis L., Seelenbinder J. (2016). Nondestructive
Handheld Fourier Transform Infrared (FT-IR) Analysis of Spectroscopic
Changes and Multivariate Modeling of Thermally Degraded Plain Portland
Cement Concrete and Its Slag and Fly Ash-Based Analogs. Appl. Spectrosc..

[4] Haas J., Mizaikoff B. (2016). Advances in Mid-Infrared Spectroscopy
for Chemical
Analysis. Annu. Rev. Anal. Chem..

[5] De
Bruyne S., Speeckaert M. M., Delanghe J. R. (2018). Applications of
Mid-Infrared Spectroscopy in the Clinical Laboratory Setting. Crit. Rev. Clin. Lab. Sci..

[6] Gautam R., Chandrasekar B., Deobagkar-Lele M., Rakshit S., Kumar B. N. V., Umapathy S., Nandi D. (2012). Identification
of Early Biomarkers
during Acetaminophen-Induced Hepatotoxicity by Fourier Transform Infrared
Microspectroscopy. PLoS One.

[7] Rafferty D. W., Koenig J. L. (2002). FTIR Imaging for the Characterization
of Controlled-Release
Drug Delivery Applications. J. Controlled Release.

[8] Diem M., Mazur A., Lenau K., Schubert J., Bird B., Miljković M., Krafft C., Popp J. (2013). Molecular Pathology *via* IR and Raman Spectral Imaging. J. Biophotonics.

[9] Baiz C. R. (2020). Vibrational Spectroscopic
Map, Vibrational Spectroscopy, and Intermolecular
Interaction. Chem. Rev..

[10] Barone V., Alessandrini S., Biczysko M., Cheeseman J. R., Clary D. C., McCoy A. B., DiRisio R. J., Neese F., Melosso M., Puzzarini C. (2021). Computational
Molecular Spectroscopy. Nat. Rev. Methods Primers.

[11] Bursch M., Mewes J.-M., Hansen A., Grimme S. (2022). Best-Practice DFT Protocols
for Basic Molecular Computational Chemistry. Angew. Chem., Int. Ed..

[12] Ditler E., Luber S. (2022). Vibrational Spectroscopy by Means of First-Principles Molecular Dynamics
Simulations. WIREs Comput. Mol. Sci..

[13] Han R., Ketkaew R., Luber S. (2022). A Concise
Review on Recent Developments
of Machine Learning for the Prediction of Vibrational Spectra. J. Phys. Chem. A.

[14] Grimme S., Bannwarth C., Shushkov P. (2017). A Robust and Accurate
Tight-Binding
Quantum Chemical Method for Structures, Vibrational Frequencies, and
Noncovalent Interactions of Large Molecular Systems Parametrized for
All Spd-Block Elements (*Z* = 1–86). J. Chem. Theory Comput..

[15] Pracht P., Grant D. F., Grimme S. (2020). Comprehensive Assessment
of GFN Tight-Binding
and Composite Density Functional Theory Methods for Calculating Gas-Phase
Infrared Spectra. J. Chem. Theory Comput..

[16] Unke O. T., Chmiela S., Sauceda H. E., Gastegger M., Poltavsky I., Schütt K. T., Tkatchenko A., Müller K.-R. (2021). Machine Learning Force Fields. Chem. Rev..

[17] Fedik N., Zubatyuk R., Kulichenko M., Lubbers N., Smith J. S., Nebgen B., Messerly R., Li Y. W., Boldyrev A. I., Barros K., Isayev O., Tretiak S. (2022). Extending Machine Learning
beyond Interatomic Potentials for Predicting Molecular Properties. Nat. Rev. Chem..

[18] Gastegger M., Schütt K. T., Müller K.-R. (2021). Machine
Learning of Solvent Effects
on Molecular Spectra and Reactions. Chem. Sci..

[19] Batatia I., Batzner S., Kovács D. P., Musaelian A., Simm G. N. C., Drautz R., Ortner C., Kozinsky B., Csányi G. (2025). The Design Space of E(3)-Equivariant
Atom-Centred Interatomic
Potentials. Nat. Mach. Intell..

[20] Anstine D. M., Zubatyuk R., Isayev O. (2025). AIMNet2: A
Neural Network Potential
to Meet Your Neutral, Charged, Organic, and Elemental-Organic Needs. Chem. Sci..

[21] Gastegger M., Behler J., Marquetand P. (2017). Machine Learning Molecular Dynamics
for the Simulation of Infrared Spectra. Chem.
Sci..

[22] Xu N., Rosander P., Schäfer C., Lindgren E., Österbacka N., Fang M., Chen W., He Y., Fan Z., Erhart P. (2024). Tensorial Properties via the Neuroevolution
Potential
Framework: Fast Simulation of Infrared and Raman Spectra. J. Chem. Theory Comput..

[23] Pracht P., Pillai Y., Kapil V., Csányi G., Gönnheimer N., Vondrák M., Margraf J. T., Wales D. J. (2024). Efficient
Composite Infrared Spectroscopy: Combining the Double-Harmonic Approximation
with Machine Learning Potentials. J. Chem. Theory
Comput..

[24] Joll K., Schienbein P., Rosso K. M., Blumberger J. (2024). Machine Learning
the Electric Field Response of Condensed Phase Systems Using Perturbed
Neural Network Potentials. Nat. Commun..

[25] Eastman P., Behara P. K., Dotson D. L., Galvelis R., Herr J. E., Horton J. T., Mao Y., Chodera J. D., Pritchard B. P., Wang Y. (2023). SPICE, A Dataset of
Drug-like Molecules and Peptides
for Training Machine Learning Potentials. Sci.
Data.

[26] Smith J. S., Zubatyuk R., Nebgen B., Lubbers N., Barros K., Roitberg A. E., Isayev O., Tretiak S. (2020). The ANI-1ccx and ANI-1x
Data Sets, Coupled-Cluster and Density Functional Theory Properties
for Molecules. Sci. Data.

[27] Ganscha S., Unke O. T., Ahlin D., Maennel H., Kashubin S., Müller K.-R. (2025). The QCML Dataset, Quantum Chemistry
Reference Data
from 33.5M DFT and 14.7B Semi-Empirical Calculations. Sci. Data.

[28] Kabylda A., Frank J. T., Suárez-Dou S., Khabibrakhmanov A., Medrano Sandonas L., Unke O. T., Chmiela S., Müller K.-R., Tkatchenko A. (2025). Molecular simulations with a pretrained
neural network
and universal pairwise force fields. J. Am.
Chem. Soc..

[29] Seung, H. S. ; Opper, M. ; Sompolinsky, H. , Query by Committee. In Proceedings of the fifth annual workshop on Computational learning theory; Association for Computing Machinery: 1992, 287–294.

[30] Settles, B. Active Learning Literature Survey; University of Wisconsin-Madison Department of Computer Sciences, 2009.

[31] Zhang D., Liu X., Zhang X., Zhang C., Cai C., Bi H., Du Y., Qin X., Peng A., Huang J. (2024). DPA-2:
A Large Atomic Model as a Multi-Task Learner. Npj Comput. Mater..

[32] Csányi G., Albaret T., Payne M. C., De Vita A. (2004). “Learn on the
Fly”: A Hybrid Classical and Quantum-Mechanical Molecular Dynamics
Simulation. Phys. Rev. Lett..

[33] Batatia, I. ; Kovacs, D. P. ; Simm, G. ; Ortner, C. ; Csanyi, G. , MACE: Higher Order Equivariant Message Passing Neural Networks for Fast and Accurate Force Fields. In Advances in Neural Information Processing Systems; Curran Associates, Inc.: 2022, 11423–11436.

[34] Kovács D. P., Moore J. H., Browning N. J., Batatia I., Horton J. T., Pu Y., Kapil V., Witt W. C., Magdău I.-B., Cole D. J., Csányi G. (2025). MACE-OFF:
Short-Range Transferable
Machine Learning Force Fields for Organic Molecules. J. Am. Chem. Soc..

[35] Tang Z., Bromley S. T., Hammer B. (2023). A Machine Learning Potential for
Simulating Infrared Spectra of Nanosilicate Clusters. J. Chem. Phys..

[36] Bhatia N., Rinke P., Krejči O. (2025). Leveraging
Active Learning-Enhanced
Machine-Learned Interatomic Potential for Efficient Infrared Spectra
Prediction. Npj Comput. Mater..

[37] Baerends E. J., Aguirre N. F., Austin N. D., Autschbach J., Bickelhaupt F. M., Bulo R., Cappelli C., van Duin A. C. T., Egidi F., Fonseca Guerra C. (2025). The Amsterdam Modeling
Suite. J. Chem. Phys..

[38] Smith J. S., Isayev O., Roitberg A. E. (2017). ANI-1: An Extensible Neural Network
Potential with DFT Accuracy at Force Field Computational Cost. Chem. Sci..

[39] Versluis L., Ziegler T. (1988). The Determination of Molecular Structures by Density
Functional Theory. The Evaluation of Analytical Energy Gradients by
Numerical Integration. J. Chem. Phys..

[40] Fan L., Ziegler T. (1991). Optimization of Molecular
Structures by Self-Consistent
and Nonlocal Density-Functional Theory. J. Chem.
Phys..

[41] Swart M., Matthias Bickelhaupt F. (2006). Optimization
of Strong and Weak Coordinates. Int. J. Quantum
Chem..

[42] Porezag D., Pederson M. R. (1996). Infrared Intensities
and Raman-scattering Activities
within Density-Functional Theory. Phys. Rev.
B.

[43] Neugebauer J., Reiher M., Kind C., Hess B. A. (2002). Quantum Chemical
Calculation of Vibrational Spectra of Large Molecules—Raman
and IR Spectra for Buckminsterfullerene. J.
Comput. Chem..

[44] Nosé S. (1984). A Unified
Formulation of the Constant Temperature Molecular Dynamics Methods. J. Chem. Phys..

[45] Hoover W. G. (1985). Canonical
Dynamics: Equilibrium Phase-Space Distributions. Phys. Rev. A.

[46] Landrum, G. ; Kelley, B. ; Tosco, P. ; Vianello, R. ; Dalke, A. ; Turk, S. ; Swain, M. ; Cole, B. ; Pahl, A. ; Fuller, P. , . Rdkit/Rdkit: 2017_09_1 (Q3 2017) Release; Zenodo, 2017.

[47] Schütt, K. ; Unke, O. ; Gastegger, M. , Equivariant Message Passing for the Prediction of Tensorial Properties and Molecular Spectra. In Proceedings of the 38th International Conference on Machine Learning; PMLR: 2021, 9377–9388.

[48] Zou Z., Zhang Y., Liang L., Wei M., Leng J., Jiang J., Luo Y., Hu W. (2023). A Deep Learning Model
for Predicting Selected Organic Molecular Spectra. Nat. Comput. Sci..

[49] Zhang Y., Jiang B. (2023). Universal Machine Learning for the
Response of Atomistic Systems
to External Fields. Nat. Commun..

[50] Li J., Knijff L., Zhang Z.-Y., Andersson L., Zhang C. (2025). PiNN: Equivariant Neural Network Suite for Modeling Electrochemical
Systems. J. Chem. Theory Comput..

[51] Batatia I., Benner P., Chiang Y., Elena A. M., Kovács D. P., Riebesell J., Advincula X. R., Asta M., Avaylon M., Baldwin W. J. (2025). A Foundation Model for Atomistic Materials
Chemistry. J. Chem. Phys..

[52] Te
Velde G., Bickelhaupt F. M., Baerends E. J., Fonseca
Guerra C., Van Gisbergen S. J. A., Snijders J. G., Ziegler T. (2001). Chemistry
with ADF. J. Comput. Chem..

[53] Xu X., Goddard W. A. (2004). The X3LYP Extended
Density Functional for Accurate
Descriptions of Nonbond Interactions, Spin States, and Thermochemical
Properties. Proc. Natl. Acad. Sci. U. S. A..

[54] Schütt K. T., Hessmann S. S., Gebauer N. W., Lederer J., Gastegger M. (2023). SchNetPack
2.0: A neural network toolbox for atomistic machine learning. J. Chem. Phys..

[55] Kuhn H. W. (1955). The Hungarian
Method for the Assignment Problem. Nav. Res.
Logist. Q.

[56] Konkoli Z., Cremer D. (1998). A New Way of Analyzing Vibrational Spectra. III. Characterization
of Normal Vibrational Modes in Terms of Internal Vibrational Modes. Int. J. Quantum Chem..

[57] Esch B. V. D., Peters L. D. M., Sauerland L., Ochsenfeld C. (2021). Quantitative
Comparison of Experimental and Computed IR-Spectra Extracted from
Ab Initio Molecular Dynamics. J. Chem. Theory
Comput..

[58] Kraka E., Quintano M., La Force H. W., Antonio J. J., Freindorf M. (2022). The Local
Vibrational Mode Theory and Its Place in the Vibrational Spectroscopy
Arena. J. Phys. Chem. A.

[59] Jacob C. R., Reiher M. (2009). Localizing Normal Modes
in Large Molecules. J. Chem. Phys..

[60] Kraka E., Zou W., Tao Y. (2020). Decoding Chemical Information from Vibrational Spectroscopy
Data: Local Vibrational Mode Theory. WIREs Comput.
Mol. Sci..

[61] Lavine B., Almirall J., Muehlethaler C., Neumann C., Workman J. (2020). Criteria for
Comparing Infrared Spectra – A Review of the Forensic and Analytical
Chemistry Literature. Forensic Chem..

[62] Western C. M., Billinghurst B. E. (2019). Automatic
and Semi-Automatic Assignment and Fitting
of Spectra with PGOPHER. Phys. Chem. Chem. Phys..

[63] Jung G., Jung S. G., Cole J. M. (2023). Automatic
Materials Characterization
from Infrared Spectra Using Convolutional Neural Networks. Chem. Sci..

[64] Tomellini S. A., Hartwick R. A., Stevenson J. M., Woodruff H. B. (1984). Automated Rule Generation
for the Program for the Analysis of Infrared Spectra (Pairs). Anal. Chim. Acta.

[65] Linder R., Nispel M., Häber T., Kleinermanns K. (2005). Gas-Phase
FT-IR-Spectra of Natural Amino Acids. Chem.
Phys. Lett..

[66] Linder R., Seefeld K., Vavra A., Kleinermanns K. (2008). Gas Phase
Infrared Spectra of Nonaromatic Amino Acids. Chem. Phys. Lett..

