# Supplementary material for: Flexible On-the-Fly Active Learning for Molecular Infrared Spectra
Source: J Chem Theory Comput. 2026 Sep 28;22(19):10309–23. doi: 10.1021/acs.jctc.6c00756 (PMC13671170; doi:10.1021/acs.jctc.6c00756)
Supplement: Supplementary file 1 [file ct6c00756_si_001.pdf]

# Flexible on-the-fly active learning for molecular infrared spectra

Giulio Benedini,[1,2,*] Matti Hellström,[1,†] and Lucas Visscher[2,‡]

[1] *Software for Chemistry & Materials BV, De Boelelaan 1109, 1081 HV Amsterdam, The Netherlands*
[2] *Vrije Universiteit Amsterdam, De Boelelaan 1108, 1081 HZ Amsterdam, The Netherlands*

(Dated: September 25, 2026)

## Supporting Information

Supporting Information includes a schematic overview of the active-learning workflow in Figure S1; AMS settings for the calculations in Tables S1, S2, and S3; details of the duplicate-removal workflow in Section I; and a comparison of configurational-space coverage across the three *als*-MT runs in Section II, including the joint-distribution and $f_{\mathrm{max}}$-distribution comparisons in Figures S2 and S3. Section III and Figure S4 compare the energy and maximum atomic-force magnitude of newly acquired als-MT run #1 frames with earlier data. Figure S5 presents reference infrared spectra for the 20 amino-acid test systems, and Table S4 reports cosine-similarity values for partial vibrational densities of states for the baseline, als-ST, and als-MT models. Figures S9, S10, and S11 show MD-based IR spectra for the *test*-20AAMD set, comparing als-ST and als-MT across three independent active-learning runs. Figures S6, S7, and S8 show Boltzmann-averaged infrared spectra for the *test*-20AAC conformer set, comparing the final als-ST and als-MT models against DFT reference spectra across the same three runs. In Figure S12 we perform the same experiment as done previously by Esch et al. [1] for comparing different similarity measures. We chose the cosine, and from Figure S12 it performs similarly to the Pearson correlation coefficient, that is the chosen one in Ref. [1]. Using the cosine similarity allows us to compare results with other relevant work done in the same field [2] (called match score in that paper). Finally, Figure S13 presents Boltzmann-weighted IR spectra of the 20 amino acids, comparing REF and cMACE-EFD ensembles at different temperatures (300, 500, and 700 K). The data sets generated in this paper are available at zenodo[3], while the project-specific scripts are available at github[4].

## LIST OF FIGURES

S1 The generic workflow for active learning. In red the retraining step of the cMLIP (cMLIP=committee of machine learning interatomic potentials). In yellow the run step which checks the reliability of the cMLIP and selects frames for later re-training. In blue the data set of the active atomistic systems. In violet the labelling step, usually at the DFT level of theory. Finally, in green the step used to evaluate the convergence of the active learning loop step. . . . . S2

S2 Distributional comparison of the three *als*-MT training datasets in the joint space of maximum atomic-force magnitude, $f_{\mathrm{max}}$, and mass-weighted radius of gyration. The top panels show probability-normalized two-dimensional histograms on a common grid, with colour indicating the fraction of training structures in each bin. The bottom panel shows an average-linkage dendrogram based on the cosine distance between the flattened density grids. Runs #1 and #3 are most similar in this representation, whereas run #2 is more distinct. . . . . S7

S3 Comparison of the $f_{\mathrm{max}}$ distributions for the *als*-MT training datasets through active-learning iteration 3. The top panels show normalized histograms constructed using common bin edges. The bottom panel shows an average-linkage dendrogram based on the cosine distance between the normalized distributions, grouping runs #1 and #3 more closely than run #2. . . . . S8

S4 Energy above the molecule-specific minimum versus maximum atomic-force magnitude for Arginine-L and Proline-L in MT run #1. Each panel compares the frames newly acquired in the indicated active-learning iteration (red) with all earlier frames for the same two molecules (blue). Circular and cross markers denote training and validation frames, respectively. The energy is normalized per atom, and the axes are shared across panels to allow direct comparison between iterations. . . . . S9

S5 Infrared spectra for the 20 amino acids for two test sets: *test*-20AA and *test*-20AAC. *test*-20AA are gas phase spectra calculated with ADF within the double harmonic approximation with the GGA-XLYP functional. On the right Boltzmann averaged infrared spectra for the same set of molecules (at 300 K, see main text for details on the conformer selection). . . . . S10

* g.benedini@vu.nl
† hellstrom@scm.com
‡ l.visscher@vu.nl

S6 Boltzmann-averaged infrared spectra (at 300 K, see main text for details on the conformer selection) from the *test*-20AAC set: (a) *als*-ST, (b) *als*-MT for run #1. . . . . . . . . . . . . . . . . . . . . . . . . . . . . . . . . . . . . . . S11
S7 Boltzmann-averaged (at 300 K, see main text for details on the conformer selection) infrared spectra from the *test*-20AAC set: (a) *als*-ST, (b) *als*-MT for run #2. . . . . . . . . . . . . . . . . . . . . . . . . . . . . . . . . . . . . . . S12
S8 Boltzmann-averaged infrared spectra (at 300 K, see main text for details on the conformer selection) from the *test*-20AAC set: (a) *als*-ST, (b) *als*-MT for run #3. . . . . . . . . . . . . . . . . . . . . . . . . . . . . . . . . . . . . . . S13
S9 MD-based infrared spectra at 300 K from the *test*-20AAMD set: (a) *als*-ST, (b) *als*-MT for run #1. . . S14
S10 MD-based infrared spectra at 300 K from the *test*-20AAMD set: (a) *als*-ST, (b) *als*-MT for run #2. . . S15
S11 MD-based infrared spectra at 300 K from the *test*-20AAMD set: (a) *als*-ST, (b) *als*-MT for run #3. . . S16
S12 Visualization of different manipulations and the corresponding behaviour of the Pearson and Spearman correlation coefficients, the cosine similarity score, and the Wasserstein distance, inspired by the work done by Esch et al. [1]. Following description is taken from Ref. [1]: "(a,b) Effect of intensity change of the entire reference function and a selected peak, both without subsequent normalization. (c,d) Effect of gradually increasing peak displacement on the similarity measures. (d) Only one of the peaks is shifted. (e,f) Effect of peak broadening while normalizing the functions from zero to one and normalizing by the standard deviation, respectively." . . . . . . . . . . . . . . . . . . . . . . . . . . . . . . . . . . . . . . . . . . . . . . S17
S13 Boltzmann-weighted IR spectra for the 20 amino acids at 300, 500, and 700 K, comparing REF and cMACE-EFD ensembles, for the als-MT run #1. . . . . . . . . . . . . . . . . . . . . . . . . . . . . . . . . . . . . . . . . . . . . S18

## LIST OF TABLES

S1 AMS settings for the DFT, MD, geometry optimization and conformer runs. . . . . . . . . . . . . . . . . . . . . . . . S3
S2 AMS settings for the MD inital run, geometry optimization and conformer runs. . . . . . . . . . . . . . . . . . . . S4
S3 MACE yaml settings. . . . . . . . . . . . . . . . . . . . . . . . . . . . . . . . . . . . . . . . . . . . . . . . . . . . . . . . . . . . . . . . . S5
S4 Similarity between partial vibrational density of states. Cosine values are reported per Atom Type, where the hydrogens are labelled differently based on the atom they are bonded to. . . . . . . . . . . . . . . . . . S11

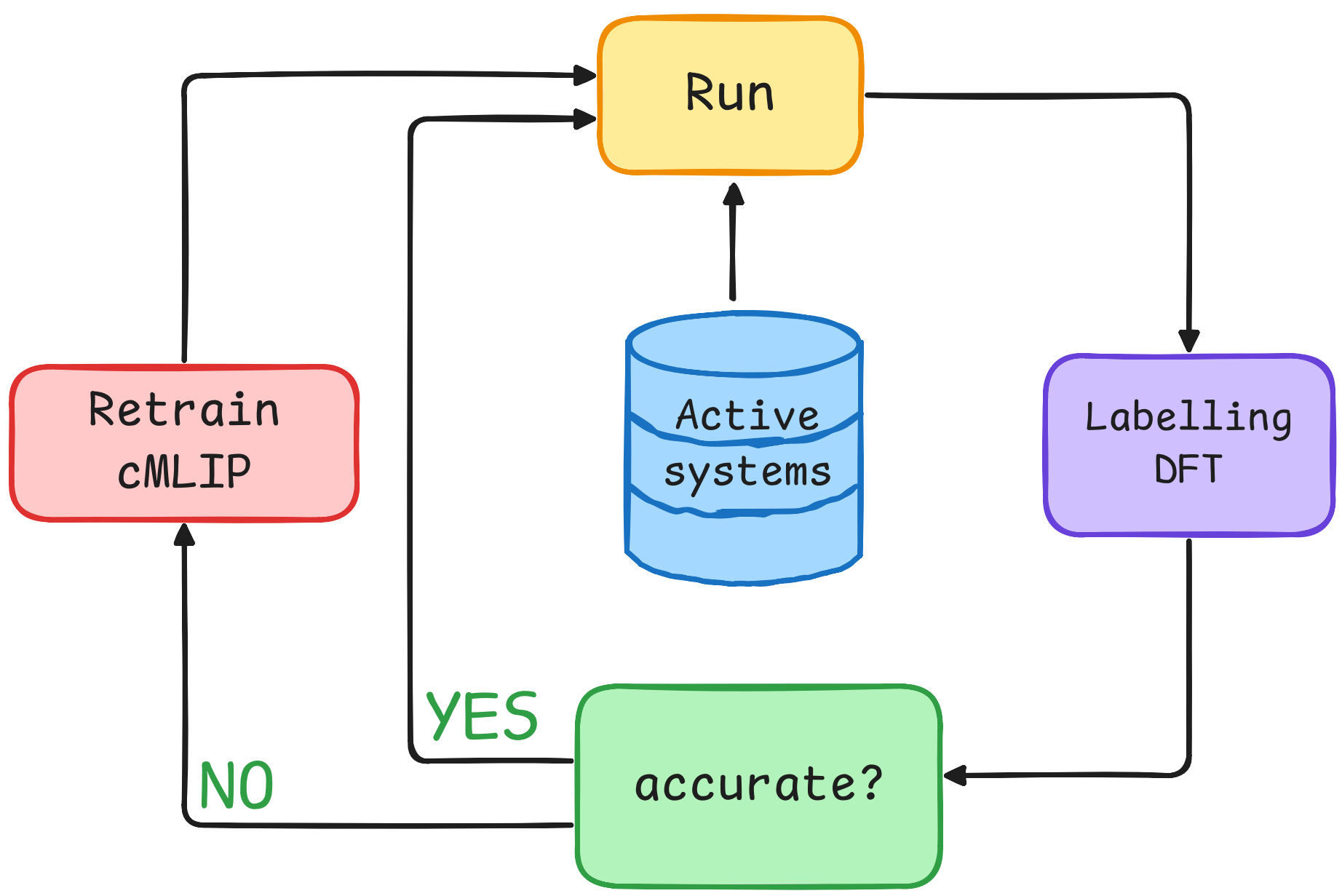


FIG. S1. The generic workflow for active learning. In red the retraining step of the cMLIP (cMLIP=committee of machine learning interatomic potentials). In yellow the run step which checks the reliability of the cMLIP and selects frames for later re-training. In blue the data set of the active atomistic systems. In violet the labelling step, usually at the DFT level of theory. Finally, in green the step used to evaluate the convergence of the active learning loop step.

TABLE S1. AMS settings for the DFT, MD, geometry optimization and conformer runs.

ADF settings for the DFT runs.

```
Engine ADF
  Basis
    Type DZP
  End
  XC
    GGA XLYP
  End
EndEngine
```

Settings for MD simulations for generate the test set.

```
MolecularDynamics
  InitialVelocities
    Temperature 300
    Type Random
  End
  NSteps 5000
  Thermostat
    Tau 200
    Temperature 300
    Type Berendsen
  End
  TimeStep 1.0
End
```

```
MolecularDynamics
  InitialVelocities
    File NVT_equ/ams.rkf
    Type FromFile
  End
  NSteps 10000
  Thermostat
    Tau 200
    Temperature 300
    Type NHC
  End
  TimeStep 1.0
End
```

```
MolecularDynamics
  BinLog
    DipoleMoment True
    Time True
  End
  InitialVelocities
    Type Input
    Values  # From NVT_prod/ams.rkf
        ...
    End
  End
  NSteps 100000
  TimeStep 0.5
End
```

TABLE S2. AMS settings for the MD inital run, geometry optimization and conformer runs.

AMS settings for the initial MD run.

```
MolecularDynamics
  BinLog
    DipoleMoment True
    Time True
  End
  InitialVelocities
    Temperature 1000
    Type Random
  End
  NSteps 10000
  Thermostat
    Tau 200.0
    Temperature 1000
    Type NHC
  End
  TimeStep 0.5
End
```

AMS settings for the geometry optimization task runs.

```
GeometryOptimization
  Convergence
    Quality Good
  End
  MaxRestarts 5
  Method Quasi-Newton
  RestartDisplacement 0.05
End

Properties
  DipoleMoment True
  NormalModes True
End

Task GeometryOptimization
```

AMS settings for the conformer task runs.

```
Generator
  Method RDKit
  RDKit
    InitialNConformers 5
  End
End

Task Generate
```

TABLE S3. MACE yaml settings.


```yaml
E0s:
  1: 0
  6: 0
  7: 0
  8: 0
  16: 0
amsgrad: true
atomic_numbers: '[1, 6, 7, 8, 16]'
batch_size: 5
correlation: 3
default_dtype: float64
device: cuda
dipole_key: REF_dipole
dipole_weight: 1.0
ema: false
ema_decay: 0.99
energy_key: REF_energy
energy_weight: 100.0
error_table: EnergyDipoleRMSE
eval_interval: 5
forces_key: REF_forces
forces_weight: 5.0
foundation_model: path/to/former/file.model
loss: energy_forces_dipole
lr: 0.01
max_L: 1
max_ell: 3
max_num_epochs: 2000
model: EnergyDipolesMACE
model_dir: results
name: model
num_channels: 64
num_interactions: 2
patience: 50
r_max: 4
seed: 20597
start_swa: 100
stress_key: REF_stress
swa: true
swa_energy_weight: 1000.0
train_file: training_set.xyz
valid_batch_size: 10
valid_file: validation_set.xyz
```

## I. DUPLICATE-REMOVAL WORKFLOW

For the *als*-MT-filter strategy and the corresponding early-stopping variant, duplicate removal was applied as a *pre-filter* to the *candidate frames* before DFT labelling. The purpose of this filter is to avoid labelling several geometries that are effectively the same structure but were selected independently by different *frame getters*.

The filter first represents every candidate frame by its Cartesian XYZ coordinates. The RMSD comparison therefore uses the atom order already present in the candidate structures: atom $i$ in one frame is compared with atom $i$ in another frame. Before RMSD values are computed, the structures are rigidly aligned with the Kabsch algorithm [5]. After rotating one structure onto the other, the aligned RMSD is evaluated as

$$\mathrm{RMSD}(P, Q) = \sqrt{\frac{1}{N_{\mathrm{atoms}}} \sum_{i=1}^{N_{\mathrm{atoms}}} \|\mathbf{p}_i - \mathbf{q}_i\|^2}, \tag{S1}$$

where $\mathbf{p}_i$ and $\mathbf{q}_i$ are the aligned Cartesian coordinates of atom $i$.

In the calculations reported here, the alignment mode was set to align all candidate frames to the first candidate structure in the pool. This corresponds to choosing candidate structure 0 as the reference structure. The choice is computationally cheaper (scales linearly as the number of frames compared) than optimizing a separate Kabsch alignment for every pair of structures, because each frame is aligned once to a common reference and the pairwise RMSD matrix is then computed in that common aligned coordinate frame. The trade-off is that the resulting RMSD matrix is mildly dependent on the chosen reference structure. We used this faster setting because the threshold was intentionally strict and the filter was only intended to remove nearly identical frames, not to perform chemically exhaustive structure pruning.

The aligned RMSD matrix was passed to an agglomerative hierarchical clustering algorithm implemented through SciPy [6]. We used complete linkage, so the distance between two clusters is the largest pairwise RMSD between their members. The flat clusters were extracted with a distance criterion and a threshold of 0.05 Å. Thus, frames were treated as duplicates only when they belonged to the same complete-linkage cluster under this 0.05 Å RMSD threshold. One representative frame was retained from each cluster and passed on to the subsequent active-learning steps. This threshold is deliberately more stringent than the 0.125 Å default used by the AMS `UniqueConformersRMSD` conformer filter, and therefore removes only highly similar structures.

## II. CONFIGURATIONAL-SPACE COVERAGE ACROSS *ALS*-MT RUNS

To investigate why *als*-MT run #2 converged after fewer iterations while retaining strong spectral accuracy, we compared the training datasets from the three independent *als*-MT runs. For a consistent comparison, all datasets were truncated after active-learning iteration 3, excluding structures sampled during later iterations of runs #1 and #3. We characterized each structure using the maximum atomic-force magnitude, $f_{\max}$, and the mass-weighted radius of gyration. These descriptors probe force magnitude and global molecular compactness, respectively, but are only low-dimensional projections of the full configurational space.

The joint distributions in Figure S2 show that run #2 is modestly distinct from runs #1 and #3. Its $f_{\max}$ distribution also contains a larger fraction of low-$f_{\max}$ structures (Figure S3), consistent with more extensive sampling near local minima. Such sampling may benefit the targeted harmonic and conformer-based IR benchmarks. Because the workflow trains system-specific rather than universal models, enrichment of task-relevant configurations is an intended feature of the active-learning procedure.

This enrichment nevertheless represents a coverage trade-off: concentrating samples near low-force configurations may reduce the representation of distorted, high-force structures and therefore limit robustness outside the targeted domain. Moreover, the observed association does not establish that the low-$f_{\max}$ structures caused the faster convergence of run #2; it may reflect stochastic variation or differences along configurational degrees of freedom not captured by these descriptors. We therefore interpret the result as a dataset-distribution effect rather than a general sampling prescription.

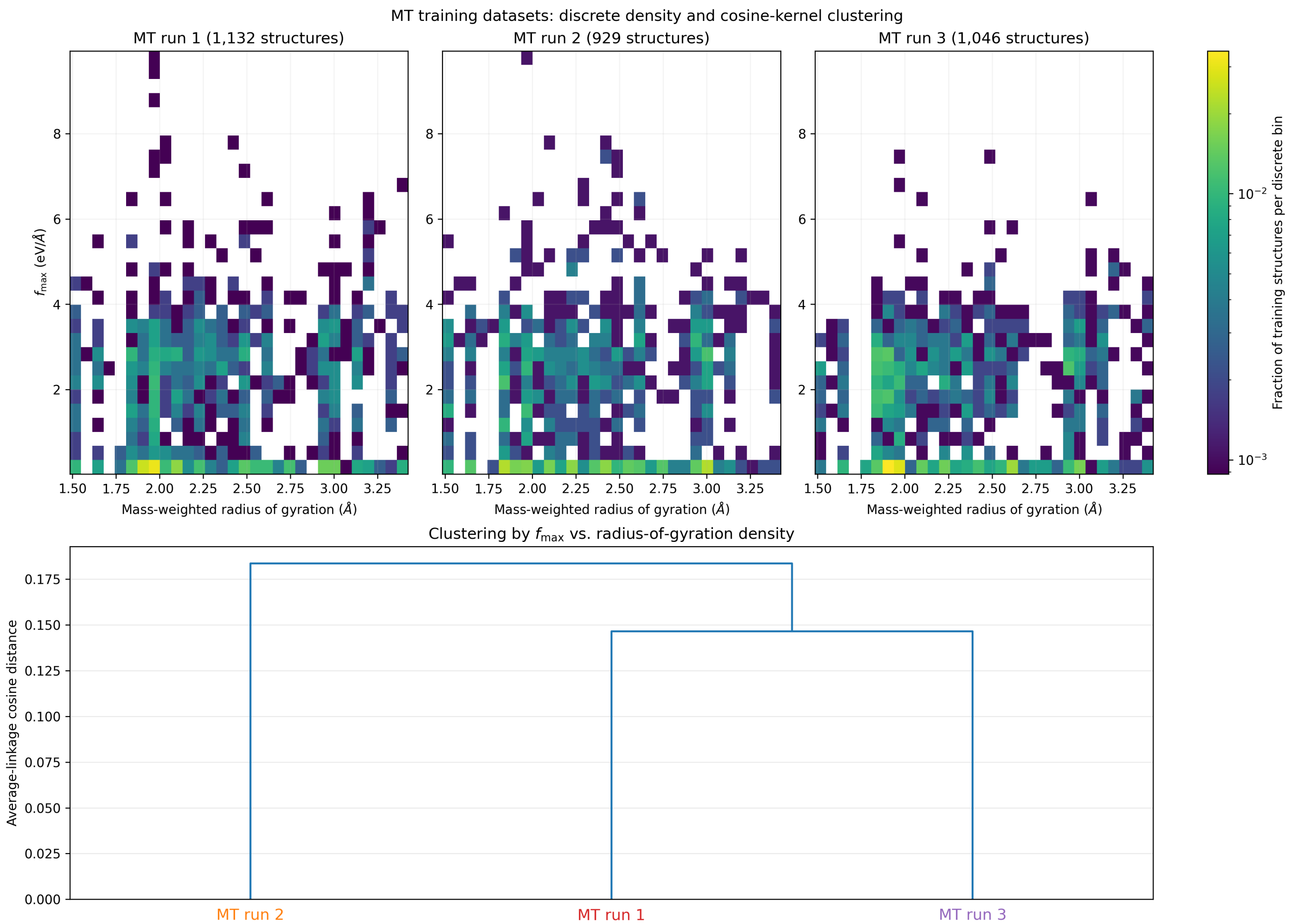


FIG. S2. Distributional comparison of the three *als*-MT training datasets in the joint space of maximum atomic-force magnitude, $f_{\max}$, and mass-weighted radius of gyration. The top panels show probability-normalized two-dimensional histograms on a common grid, with colour indicating the fraction of training structures in each bin. The bottom panel shows an average-linkage dendrogram based on the cosine distance between the flattened density grids. Runs #1 and #3 are most similar in this representation, whereas run #2 is more distinct.

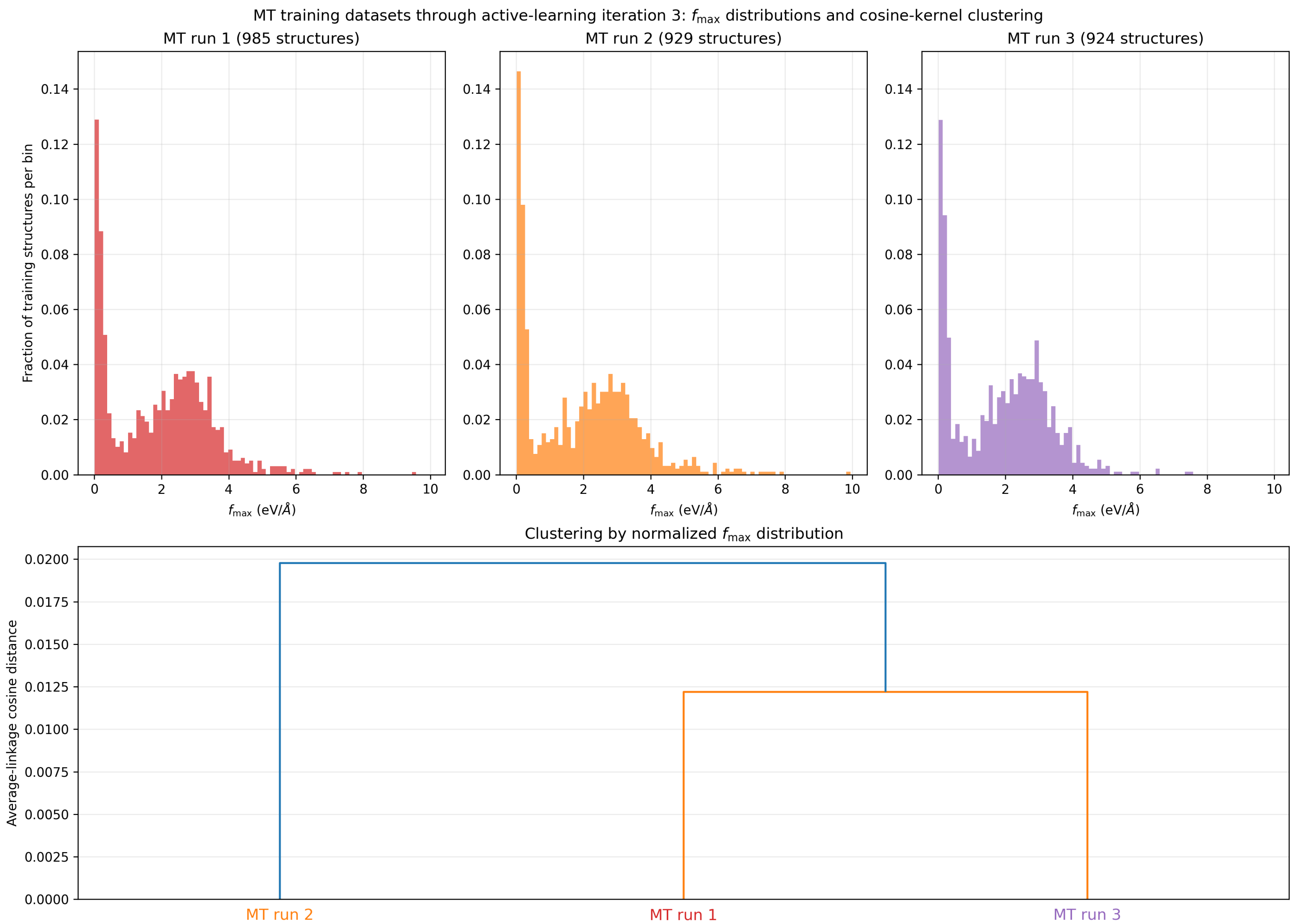


FIG. S3. Comparison of the $f_{\max}$ distributions for the *als*-MT training datasets through active-learning iteration 3. The top panels show normalized histograms constructed using common bin edges. The bottom panel shows an average-linkage dendrogram based on the cosine distance between the normalized distributions, grouping runs #1 and #3 more closely than run #2.

## III. ENERGY, FORCE COMPARISON OF NEWLY ACQUIRED ALS-MT RUN 1 FRAMES

To assess whether the non-monotonic variation in als-MT run #1 errors coincides with a change in the sampled configurations, we compared the energy and maximum atomic-force magnitude of newly acquired frames with those of earlier data. Figure S4 focuses on Arginine-L and Proline-L, the two molecules that converge most slowly in als-MT run #1. For each active-learning iteration, the prior dataset contains all frames acquired in earlier iterations for these molecules, while the newly acquired cohort contains the frames added in the iteration shown.

The newly acquired sets overlap substantially with the prior energy–force distributions. Although individual newly acquired frames occur at relatively high energies and maximum forces, comparable prior frames are also present. The visual comparison therefore does not indicate a consistent shift towards higher-energy or higher-force frames across iterations.

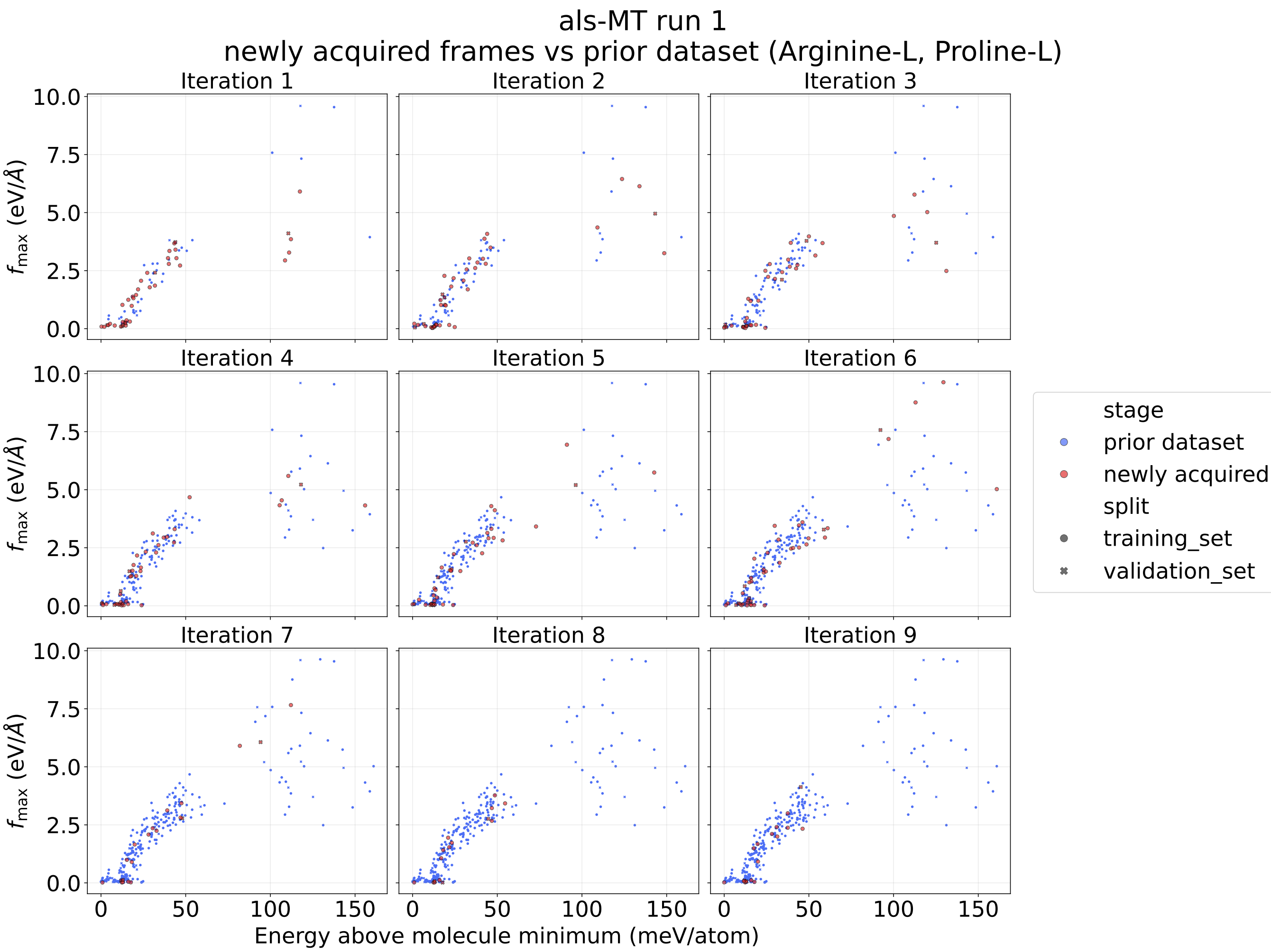


FIG. S4. Energy above the molecule-specific minimum versus maximum atomic-force magnitude for Arginine-L and Proline-L in MT run #1. Each panel compares the frames newly acquired in the indicated active-learning iteration (red) with all earlier frames for the same two molecules (blue). Circular and cross markers denote training and validation frames, respectively. The energy is normalized per atom, and the axes are shared across panels to allow direct comparison between iterations.

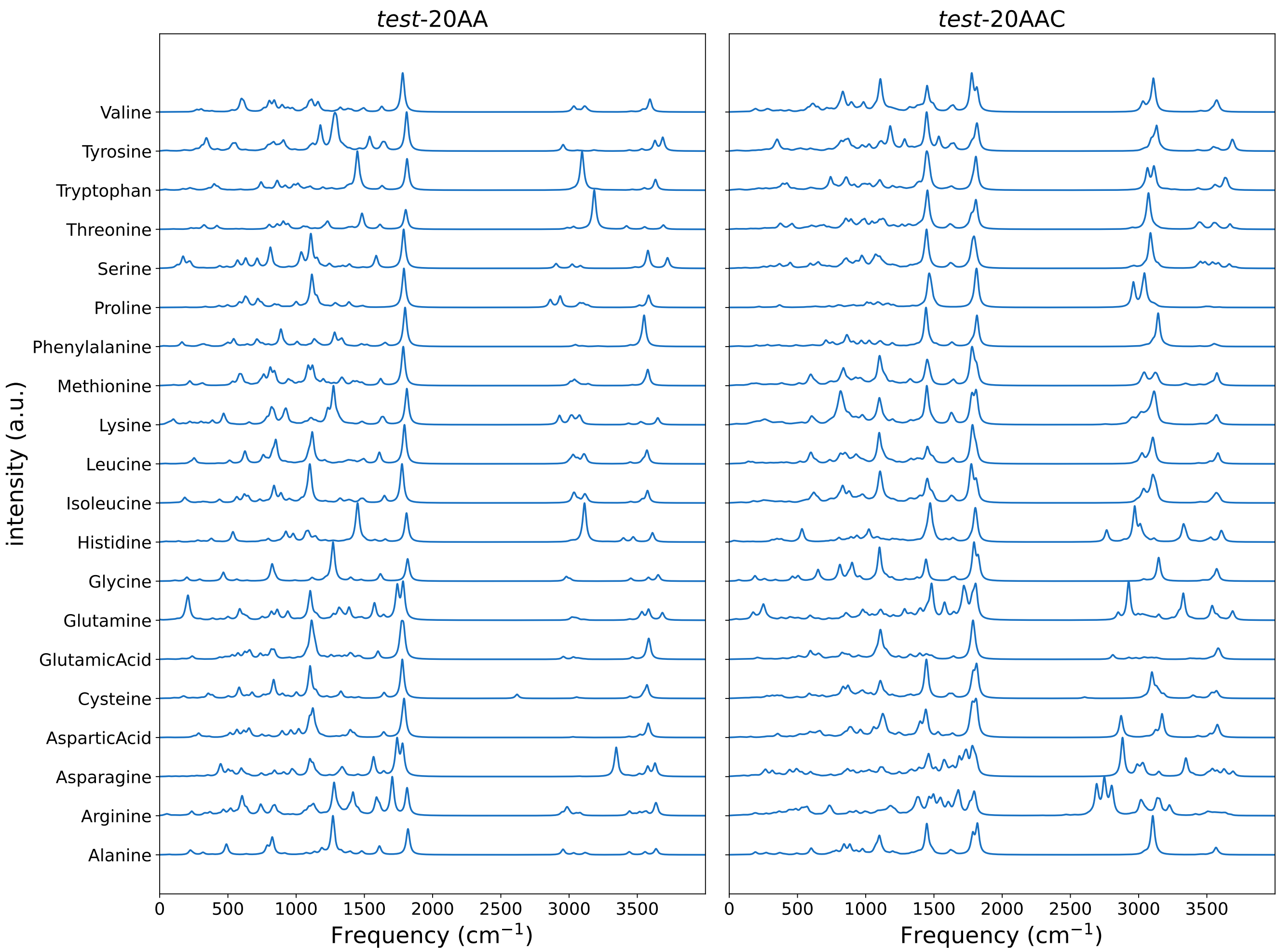


FIG. S5. Infrared spectra for the 20 amino acids for two test sets: *test*-20AA and *test*-20AAC. *test*-20AA are gas phase spectra calculated with ADF within the double harmonic approximation with the GGA-XLYP functional. On the right Boltzmann averaged infrared spectra for the same set of molecules (at 300 K, see main text for details on the conformer selection).

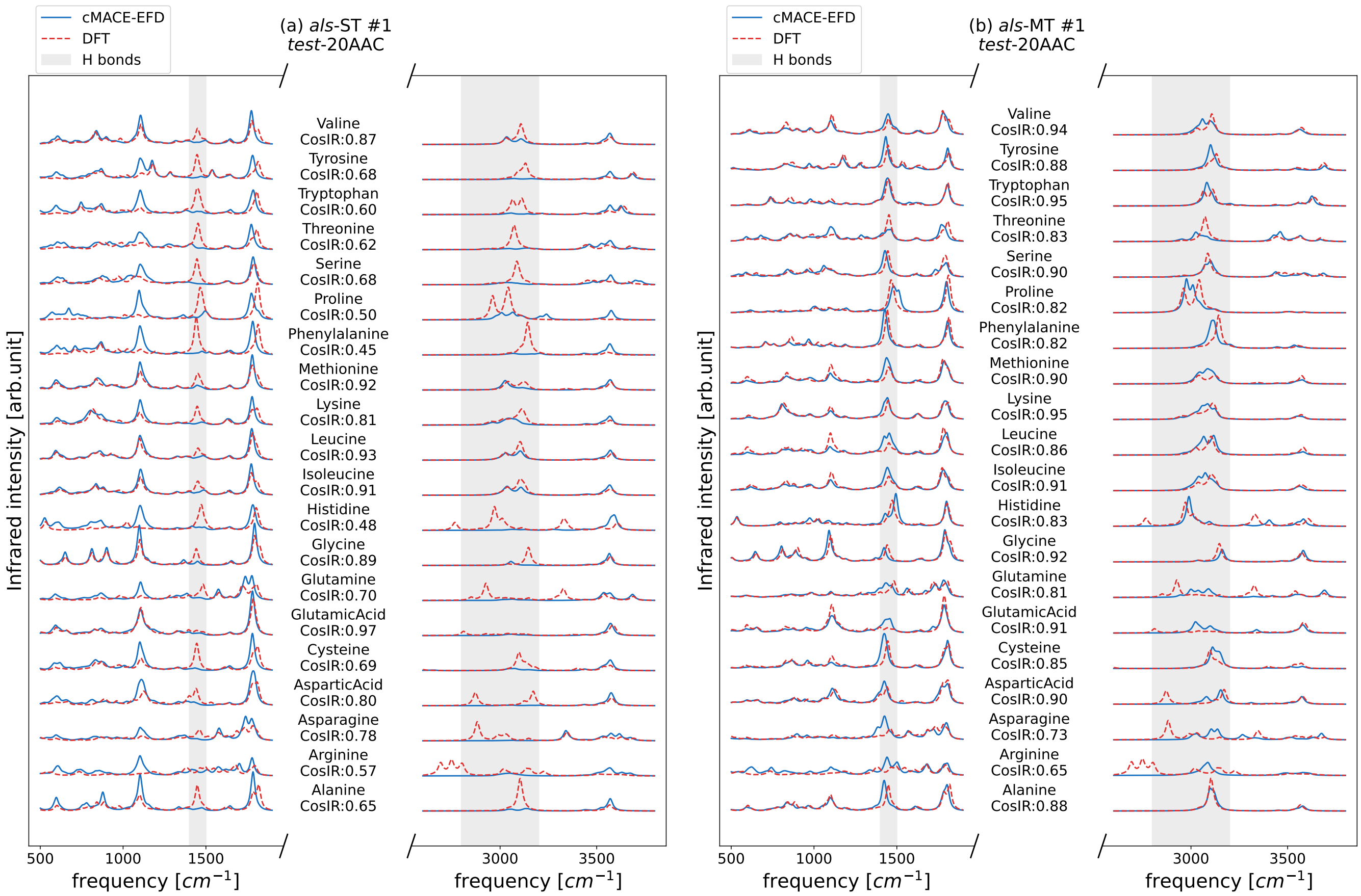


FIG. S6. Boltzmann-averaged infrared spectra (at 300 K, see main text for details on the conformer selection) from the *test*-20AAC set: (a) *als*-ST, (b) *als*-MT for run #1.

TABLE S4. Similarity between partial vibrational density of states. Cosine values are reported per Atom Type, where the hydrogens are labelled differently based on the atom they are bonded to.

| Atom Type | Baseline | *als*-MT | *als*-ST |
|---|---|---|---|
| C | 0.87 | 0.96 | 0.94 |
| HC | 0.79 | 0.92 | 0.90 |
| HN | 0.65 | 0.88 | 0.85 |
| HO | 0.65 | 0.87 | 0.83 |
| HS | 0.09 | 0.71 | 0.65 |
| N | 0.75 | 0.92 | 0.88 |
| O | 0.86 | 0.95 | 0.94 |
| S | 0.73 | 0.93 | 0.93 |

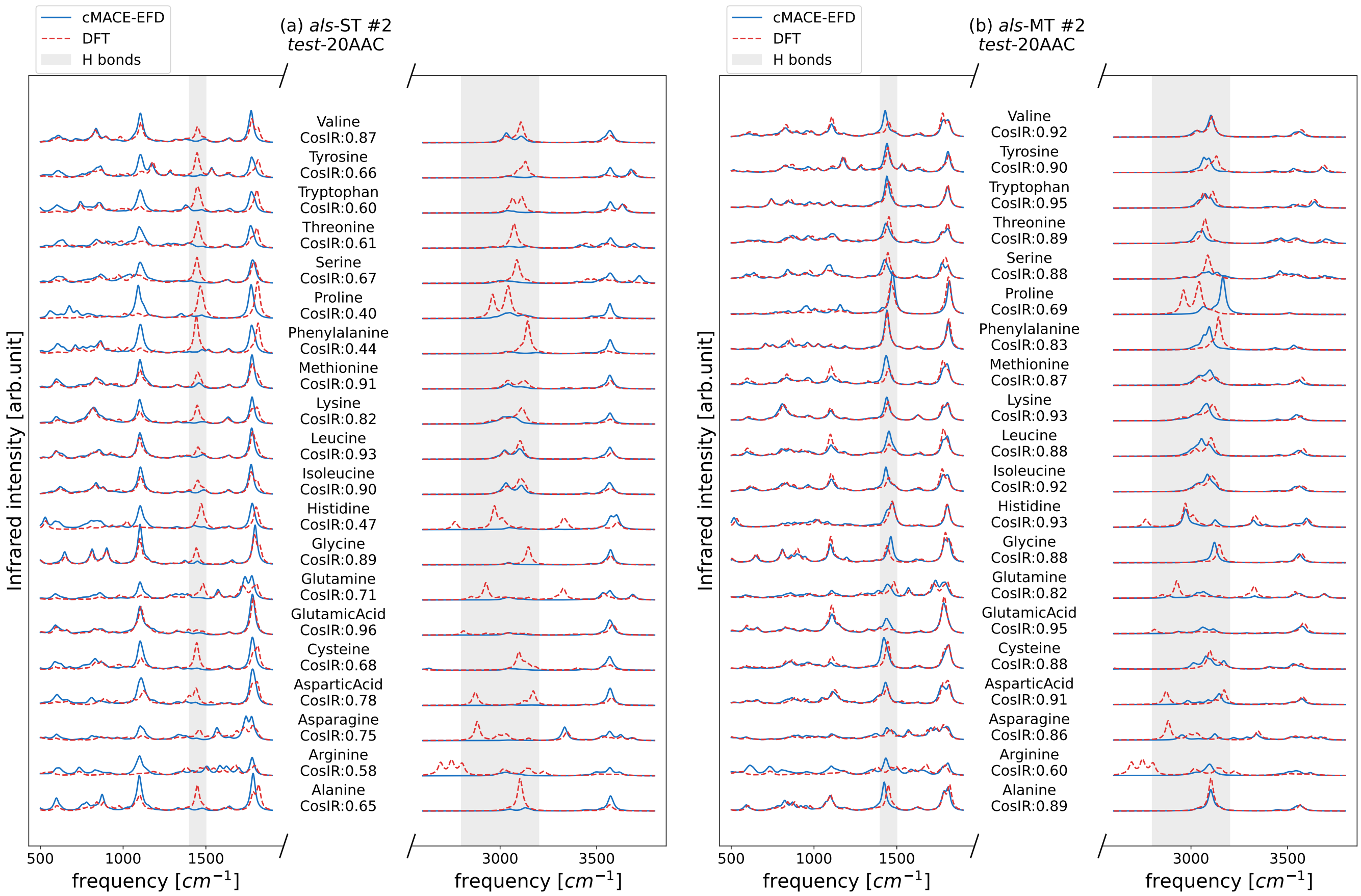


FIG. S7. Boltzmann-averaged (at 300 K, see main text for details on the conformer selection) infrared spectra from the *test*-20AAC set: (a) *als*-ST, (b) *als*-MT for run #2.

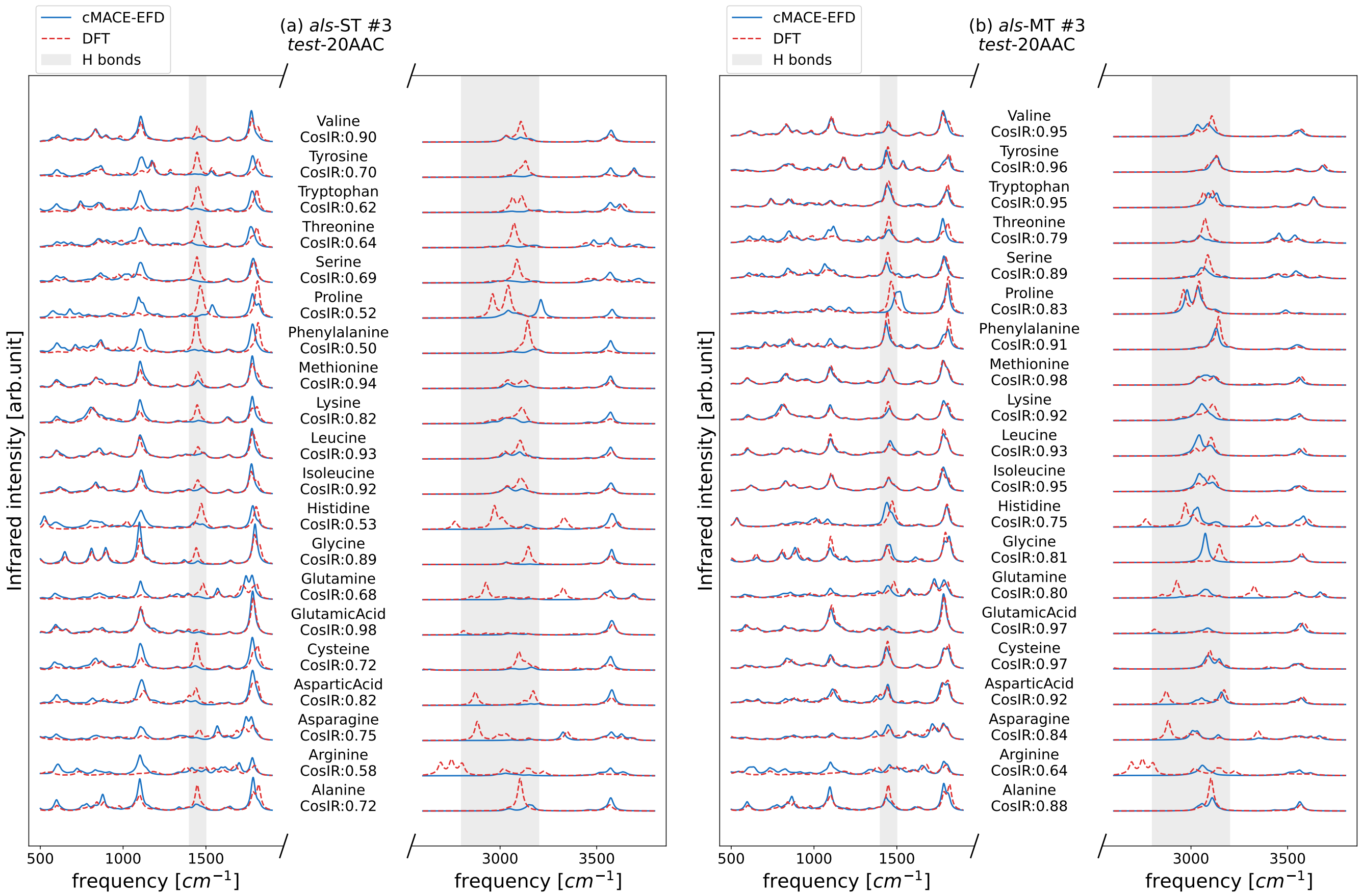


FIG. S8. Boltzmann-averaged infrared spectra (at 300 K, see main text for details on the conformer selection) from the *test*-20AAC set: (a) *als*-ST, (b) *als*-MT for run #3.

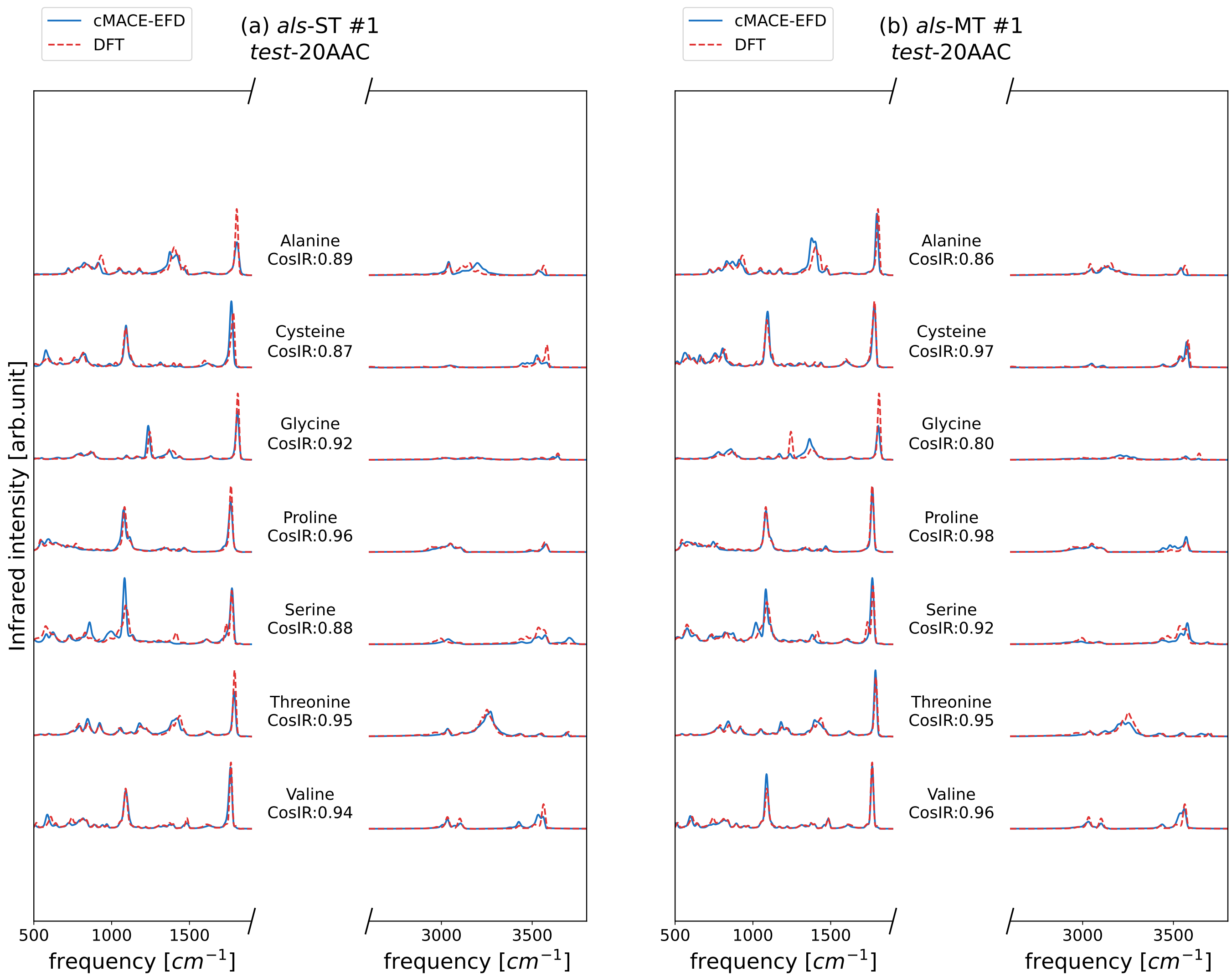


FIG. S9. MD-based infrared spectra at 300 K from the *test*-20AAMD set: (a) *als*-ST, (b) *als*-MT for run #1.

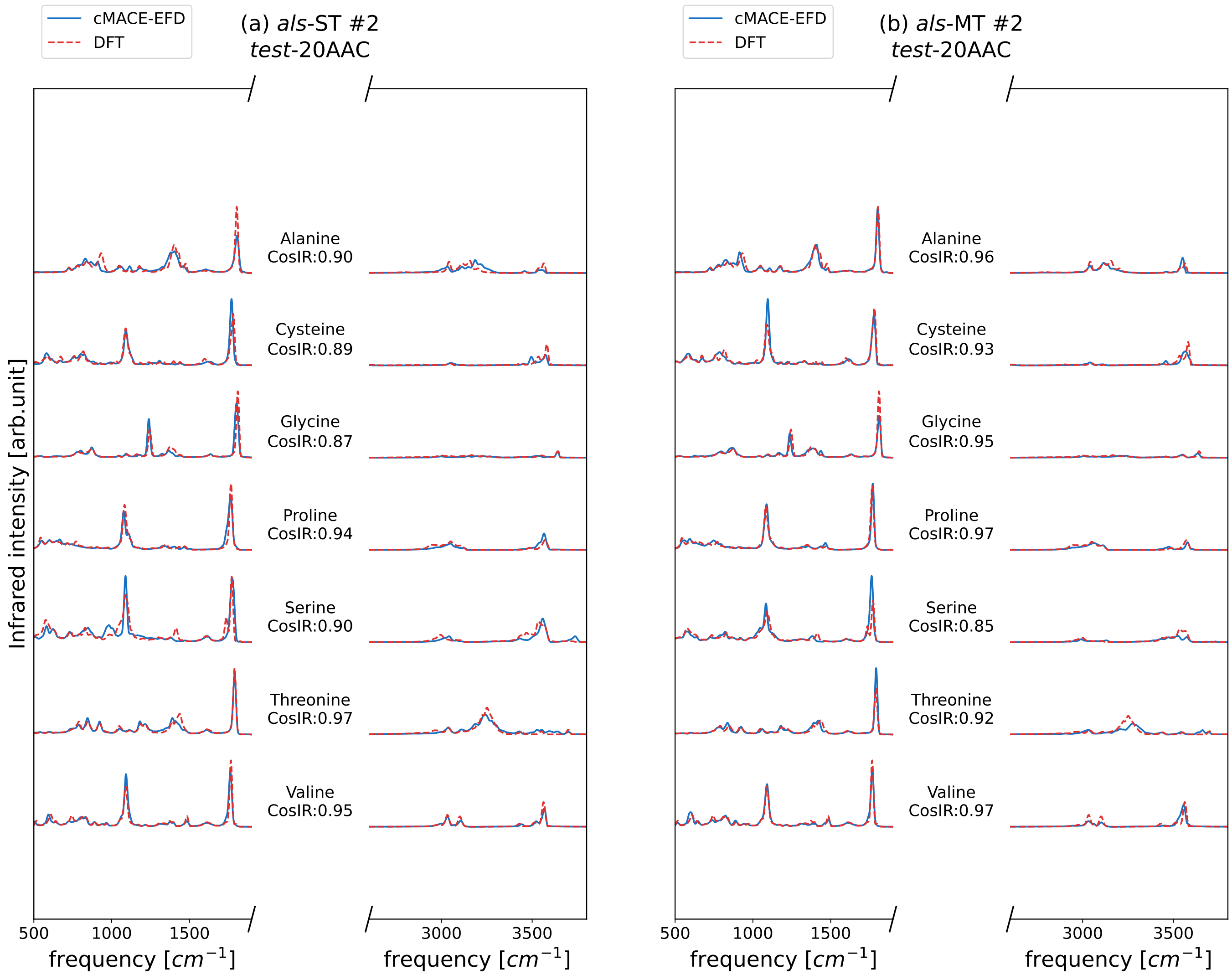


FIG. S10. MD-based infrared spectra at 300 K from the *test*-20AAMD set: (a) *als*-ST, (b) *als*-MT for run #2.

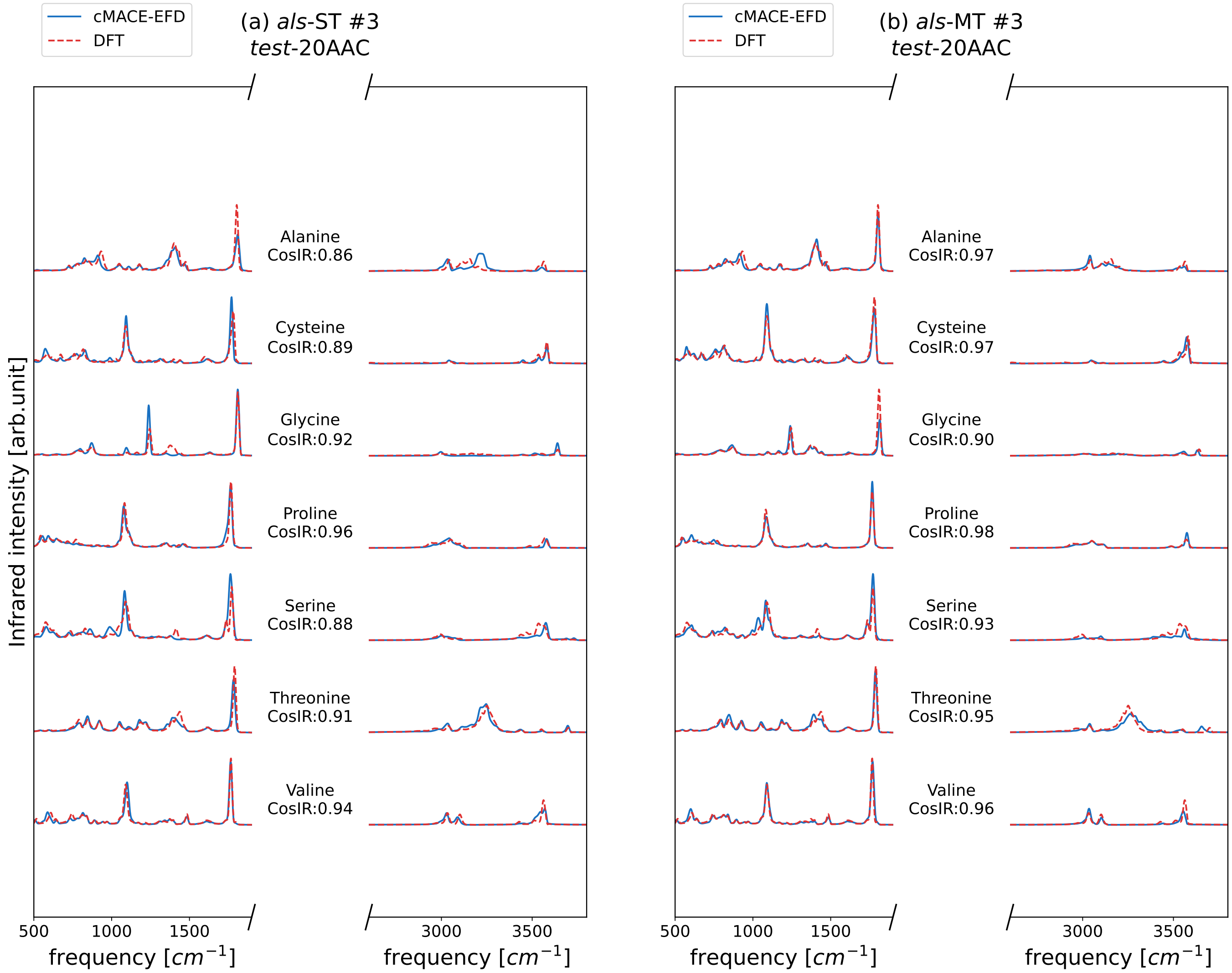


FIG. S11. MD-based infrared spectra at 300 K from the *test*-20AAMD set: (a) *als*-ST, (b) *als*-MT for run #3.

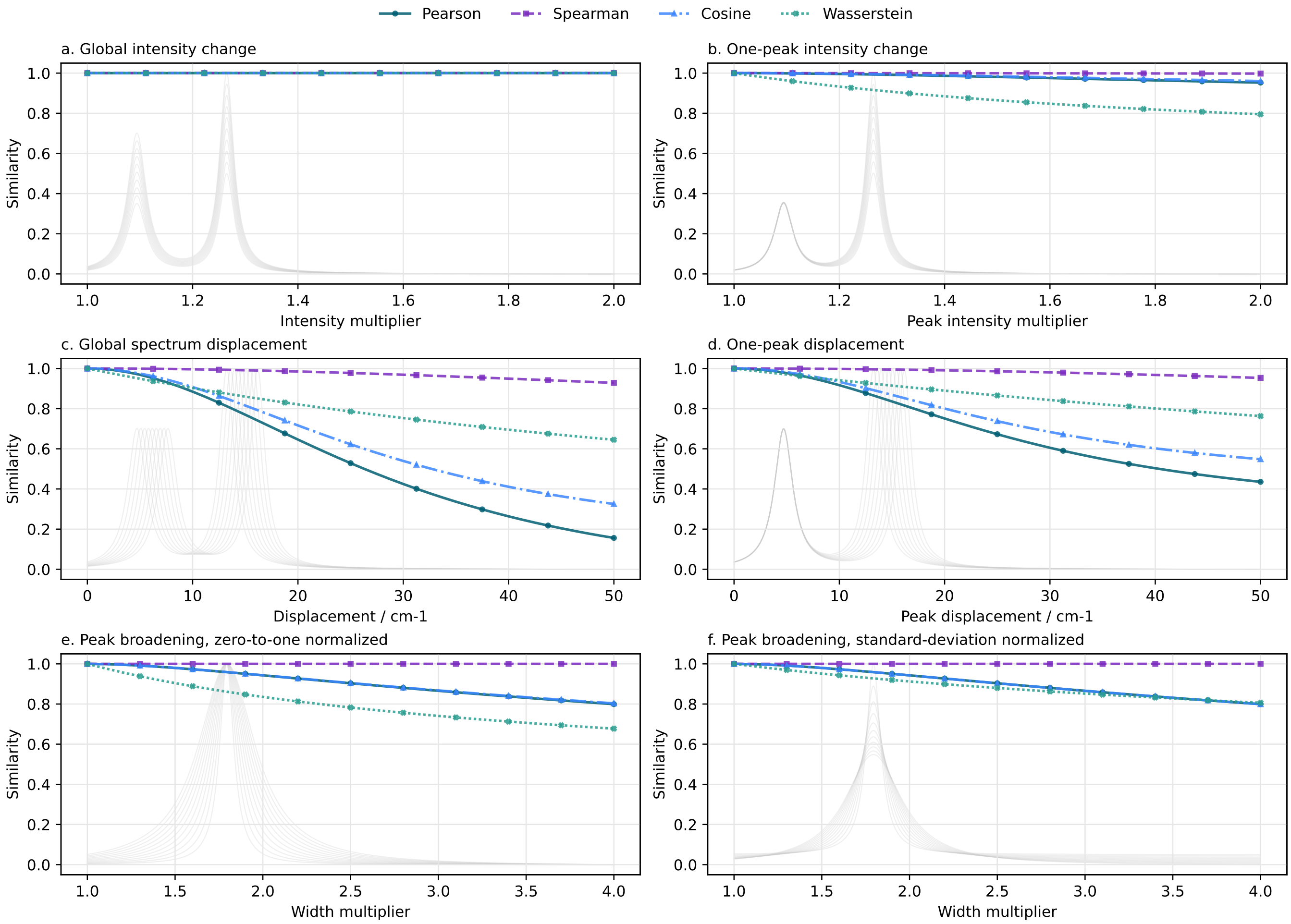


FIG. S12. Visualization of different manipulations and the corresponding behaviour of the Pearson and Spearman correlation coefficients, the cosine similarity score, and the Wasserstein distance, inspired by the work done by Esch et al. [1]. Following description is taken from Ref. [1]: "(a,b) Effect of intensity change of the entire reference function and a selected peak, both without subsequent normalization. (c,d) Effect of gradually increasing peak displacement on the similarity measures. (d) Only one of the peaks is shifted. (e,f) Effect of peak broadening while normalizing the functions from zero to one and normalizing by the standard deviation, respectively."

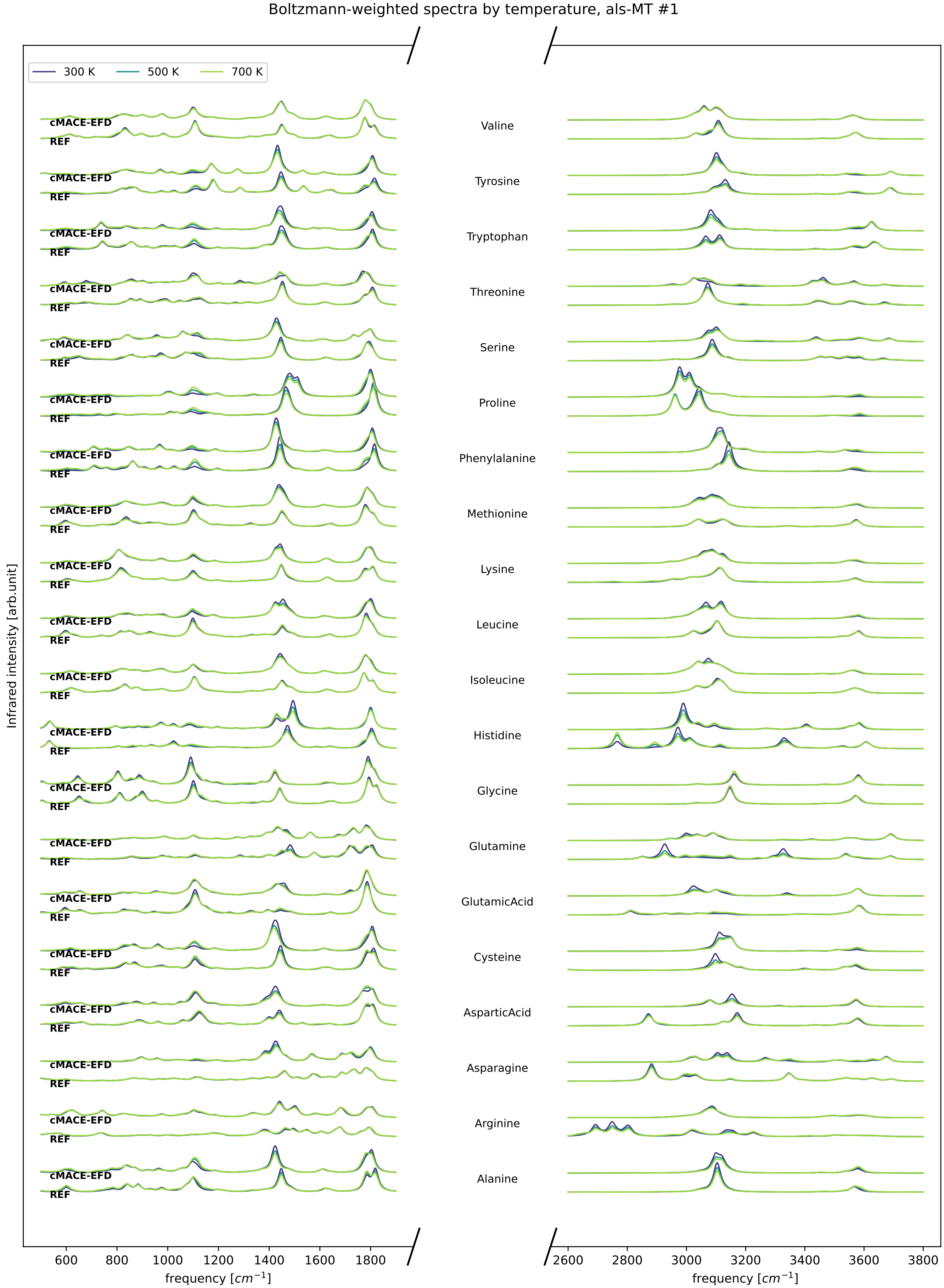


FIG. S13. Boltzmann-weighted IR spectra for the 20 amino acids at 300, 500, and 700 K, comparing REF and cMACE-EFD ensembles, for the als-MT run #1.

## IV. REFERENCES


[1] Esch, B. V. D.; Peters, L. D. M.; Sauerland, L.; Ochsenfeld, C. Quantitative Comparison of Experimental and Computed IR-Spectra Extracted from Ab Initio Molecular Dynamics. *Journal of Chemical Theory and Computation* **2021**, *17*, 985–995.

[2] Pracht, P.; Pillai, Y.; Kapil, V.; Csányi, G.; Gönnheimer, N.; Vondrák, M.; Margraf, J. T.; Wales, D. J. Efficient Composite Infrared Spectroscopy: Combining the Double-Harmonic Approximation with Machine Learning Potentials. *Journal of Chemical Theory and Computation* **2024**, *20*, 10986–11004.

[3] Benedini, G. Test sets of: "Flexible On-the-Fly Active Learning for Molecular Infrared Spectra". 2026; `https://doi.org/10.5281/zenodo.22942622`.

[4] Benedini, G. Flexible On-the-Fly Active Learning for Molecular Infrared Spectra. 2026; `https://github.com/GiulioIlBen/active_learning_for_IR_spectra`, GitHub repository, commit eb7090f9452e6e88b8de73ee30aff76875b2fceb.

[5] Kabsch, W. A Solution for the Best Rotation to Relate Two Sets of Vectors. *Foundations of Crystallography* **1976**, *32*, 922–923.

[6] Virtanen, P.; Gommers, R.; Oliphant, T. E.; Haberland, M.; Reddy, T.; Cournapeau, D.; Burovski, E.; Peterson, P.; Weckesser, W.; Bright, J.; others SciPy 1.0: fundamental algorithms for scientific computing in Python. *Nature methods* **2020**, *17*, 261–272.
